# AGAMOUS-LIKE24 controls pistil number in Japanese apricot by targeting the *KNOTTED1-LIKE* gene *KNAT2/6-a*

**DOI:** 10.1093/plphys/kiae069

**Published:** 2024-02-12

**Authors:** Yang Bai, Pengyu Zhou, Zhaojun Ni, Shahid Iqbal, Kenneth Omondi Ouma, Xiao Huang, Feng Gao, Chengdong Ma, Ting Shi, Zhihong Gao

**Affiliations:** Laboratory of Fruit Tree Biotechnology, College of Horticulture, Nanjing Agricultural University, Nanjing 210095, China; Laboratory of Fruit Tree Biotechnology, College of Horticulture, Nanjing Agricultural University, Nanjing 210095, China; Laboratory of Fruit Tree Biotechnology, College of Horticulture, Nanjing Agricultural University, Nanjing 210095, China; Horticultural Science Department, North Florida Research and Education Center, University of Florida/IFAS, Quincy, FL 32351, USA; Laboratory of Fruit Tree Biotechnology, College of Horticulture, Nanjing Agricultural University, Nanjing 210095, China; Laboratory of Fruit Tree Biotechnology, College of Horticulture, Nanjing Agricultural University, Nanjing 210095, China; Laboratory of Fruit Tree Biotechnology, College of Horticulture, Nanjing Agricultural University, Nanjing 210095, China; Laboratory of Fruit Tree Biotechnology, College of Horticulture, Nanjing Agricultural University, Nanjing 210095, China; Laboratory of Fruit Tree Biotechnology, College of Horticulture, Nanjing Agricultural University, Nanjing 210095, China; Laboratory of Fruit Tree Biotechnology, College of Horticulture, Nanjing Agricultural University, Nanjing 210095, China

## Abstract

The formation of multi-pistil flowers reduces the yield and quality in Japanese apricot (*Prunus mume*). However, the molecular mechanism underlying the formation of multi-pistil flowers remains unknown. In the current study, overexpression of *PmKNAT2/6-a*, a class I *KNOTTED1-like homeobox* (*KNOX*) member, in Arabidopsis (*Arabidopsis thaliana*) resulted in a multi-pistil phenotype. Analysis of the upstream regulators of *PmKNAT2/6-a* showed that AGAMOUS-like 24 (PmAGL24) could directly bind to the *PmKNAT2/6-a* promoter and regulate its expression. PmAGL24 also interacted with Like Heterochromatin Protein 1 (PmLHP1) to recruit lysine trimethylation at position 27 on histone H3 (H3K27me3) to regulate *PmKNAT2/6-a* expression, which is indirectly involved in multiple pistils formation in Japanese apricot flowers. Our study reveals that the PmAGL24 transcription factor, an upstream regulator of *PmKNAT2/6-a*, regulates *PmKNAT2/6-a* expression via direct and indirect pathways and is involved in the formation of multiple pistils in Japanese apricot.

## Introduction

Japanese apricot (*Prunus mume* Sieb. et Zucc.), also known as MEI, is a Rosaceae (*Prunus* L.) plant with a long history of cultivation in Asia. The Japanese apricot, which can be grown as an ornamental plant, has lovely flowers, and the fruit has a unique flavor and is rich in nutrients, making it one of the most popular fruits. The perfect flower of the Japanese apricot usually contains 1 normal developing pistil, but there are many ornamental varieties, such as ‘Dayu’ and ‘Pinzimei’, which have 3 to several pistils that can develop normally to form fruit after pollination. Although multiple pistils in 1 flower improve the ornamental value of Japanese apricot, the presence of multiple pistils in 1 flower is frequently accompanied by a decrease in fruit quality during production. Temperature, nutrition, phytohormones, calmodulin, and lignin synthesis are the most important factors influencing pistil development. The cytokinin (CTK) response factor, *type-A Arabidopsis Response Regulators* (*A-ARR*), is the target gene of miR160a/b in Japanese apricot, which is regulated by lncRNA TCONS_00032517, resulting in a substantial amount of CTK accumulation in multi-pistil Japanese apricot flowers and promoting multi-pistil determination (Wu et al. 2019). The homeobox transcription factor, the *KNOTTED1-like homeobox* (*KNOX*) gene, is a common regulator upstream of CTK and lignin anabolism (Hay and Tsiantis 2010). The classification of the KNOX gene family into Class I, Class II, and Class M KNOX subfamilies was made based on structural characteristics, phylogenetic relationships, and expression patterns. Class I KNOX genes that regulate lateral organ formation and growth are expressed in apical meristematic tissues and determine the fate of meristematic cells (Kerstetter et al. 1994, Scofield et al. 2007). Class II KNOX genes regulate the secondary cell wall, xylem, root development, and fruit ripening (Li et al. 2012a, Keren-Keiserman et al. 2022). KNATM, which is primarily expressed at the edges of mature organs and in the proximal domains of plant organ primordia, may be used to detect or generate signals related to flowering (Magnani and Hake 2008).

In Arabidopsis (*Arabidopsis thaliana*), most class I *KNOX*s act as transcription factors involved in carpelogenesis (Douglas et al. 2002). In Arabidopsis, the Class I *KNOX* genes include *STM*, *KNAT1/BREVIPEDICELLUS (BP)*, *KNAT2*, and *KNAT6* (Scofield and Murray 2006). *STM* is expressed in the inflorescence stem meristem and floral meristem tissues, whereas *BP* is expressed only in a narrow segment of the inflorescence stem meristem cells. In *bp* function-deficient *stm* mutant, the meristematic tissues lose their differentiation ability, suggesting that maintenance of the shoot apical meristem (SAM) is performed jointly by *BP* and *STM* (Byrne et al. 2003). Similar to *BP*, the *stm-2 knat6* double mutant also shows a phenotype of loss of differentiation ability of meristematic tissues, and a failure of correct cotyledon separation was also observed, suggesting a role for *KNAT6* in maintaining plant boundaries (Belles-Boix et al. 2006). *KNAT2* and *KNAT6* are the 2 most recently related class I *KNOX* genes, and *KNAT2* overexpression partially rescues the phenotype of the *stm* mutants (Dockx et al. 1995, Byrne et al. 2000). Ectopic *KNAT2* expression triggers the development of carpel-like bead-centered structures (Ragni et al. 2008), and ectopic *KNAT2* expression expands *AGAMOUS* (*AG*) expression in carpels and ovules, as well as the development of ectopic carpel structures independent of the effects of *AG* genes. KNOX proteins are hypothesized to collaborate with factors other than AG to regulate carpel development (Gallois et al. 2002). In wild-type Arabidopsis, the BELLRINGER (BELL) family member PENNYWISE (PNY) interacts with BP to restrict the expression of *KNAT6* and *KNAT2* and promote normal inflorescence growth (Ragni et al. 2008).

The class I KNOX gene *KNAT2* is involved in the regulation of the expression of some CTK synthase genes, promoting CTK accumulation and activating the CTK response (Hamant et al. 2002). Therefore, we hypothesized that *KNAT2* is an important regulator of pistil morphogenesis in plants.

The class I KNOX locus is enriched in the lysine trimethylation (H3K27me3) tag at position 27 on histone H3 modification sites and like Heterochromatin Protein 1 (LHP1), recognizing and binding to the H3K27me3 modification site (Turck et al. 2007) LHP1 is a member of the Polycomb group (PcG) in eukaryotes, which is an important member of the epistatic regulators that regulate gene expression mainly at the transcriptional level and participate in many important cellular activities and developmental processes. PcG is mainly found in Polycomb Repressive Complex 1 (PRC1) and PRC2. PRC1 can repress target gene transcription by recognizing H3K27me3, a repressive histone marker that promotes chromosome crinkling; PRC2 can catalyze H3K27me3 in specific target genes and repress gene transcription, playing an important role in gene silencing and gene regulation (Tolhuis et al. 2006, Margueron and Reinberg 2011). The *lhp1-2* single mutant and the *lhp1-2 ag-10* double mutant usually have more than 2 carpels (Liu et al. 2014), indicating that PmLHP1 is involved in the determination process of multiple pistils in plants.

Furthermore, the *Prunus mume AGAMOUS-LIKE24* (*PmAGL24*) gene is also known as the DORMANCY-ASSOCIATED*MADS6* (*PmDAM6*). When plants transition from vegetative to reproductive growth, AGL24 interacts with flowering genes, forms a complex in the SAM tissue, transfers from the cytoplasm to the nucleus, and activates its expression on the *LEAFY* promoter to complete flowering induction in Arabidopsis (Lee et al. 2008). *AGL24* is involved in flower development and functions as a floral promoter during the early stages of flower development (Gregis et al. 2006) (Shim et al. 2014). *AGL24* overexpression promotes flowering and the transformation of floral meristems to inflorescence meristems (Michaels et al. 2003). In Japanese apricot, PmAGL24 can interact with the flowering integrator SUPPRESSOR OF OVEREXPRESSION OF CO 1 (SOC1) to regulate dormancy release (Kitamura et al. 2016). *PmAGL24* was also more highly expressed in mono-pistil Japanese apricot varieties than in multi-pistil types (Wu et al. 2019), implying that *PmAGL24* may be involved in pistil number determination in Japanese apricot.

This study showed that *PmKNAT2/6-a* is a key gene involved in multi-pistil in Japanese apricot. Its upstream transcription factor, PmAGL24, can bind to the promoter of this gene directly or indirectly to regulate *PmKNAT2/6-a* expression, which is involved in Japanese apricot multiple pistils.

## Results

### The *PmKNAT2/6-a* gene is involved in multi-pistillogenesis in Japanese apricot

**Figure 1.**
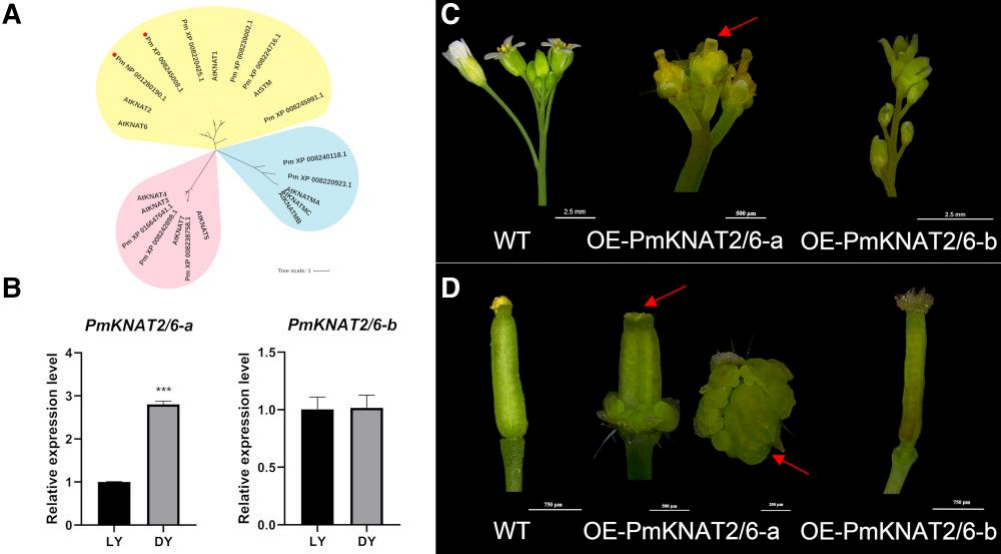
Phylogenetic and functional analyses of *PmKNAT2/6-a* and *PmKNAT2/6-b*. **A)** Phylogenetic tree of KNOX proteins from Japanese apricot and Arabidopsis. The multiple protein sequences of 11 PmKNOX and 11 AtKNOX were aligned with the MUSCLE method, and the tree was built using the maximum likelihood method using MAFFT v7.475 (bootstrap value: 1,000). The tree was categorized into 3 subfamilies: Class I (color block on top), Class II (color block at the bottom left), and Class M (color block on the bottom right). The PmKNAT2/6 proteins are indicated by red dots. **B)** Expression analysis of *PmKNAT2/6-a* and *PmKNAT2/6-b* in Japanese apricot varieties at the pistil determination stage. Error bars represent Se based on 3 biological replicates. Asterisks indicate significant differences using Student's *t*-test (***P < 0.001). **C and D)** Functional analysis of the *PmKNAT2/6-a* and *PmKNAT2/6-b*. **C)** Inflorescence of transgenic plants. **D)** The transgenic plants’ carpel is the second line. The red arrow indicates the location of multiple carpels. Images were digitally extracted for comparison. The transgenic Arabidopsis lines are OE-PmKNAT2/6-a and OE-PmKNAT2/6-b, and WT is wild-type Arabidopsis.

The KNOTTED1-like homeobox (KNOX) genes were queried in the Japanese apricot genome based on the KNOX gene family conserved structural domains KNOX1 (pfam03790), KNOX2 (pfam03791), ELK (pfam03789), and Homeobox KN (pfam05920). A phylogenetic tree with 11 candidate genes and 9 Arabidopsis KNOX genes was constructed because the function of the KNOX gene family in the model plant Arabidopsis has been extensively studied and the subfamilies have been clearly defined (Magnani and Hake 2008, Piazza et al. 2010, Zhang et al. 2022) (Fig. 1A). Comparatively, XP_008245008.1 and NP_001280190.1 belonged to the same branch as homeobox knotted-like 2 (AtKNAT2) and homeobox knotted-like 6 (AtKNAT6) in Arabidopsis, indicating functional similarity. Based on their proximity to AtKNAT2 and AtKNAT6 in the phylogenetic tree, these genes were named PmKNAT2/6-a and PmKNAT2/6-b, respectively; since overexpression of *KNAT2* in Arabidopsis expands *AGAMOUS* (*AG*) expression in carpels and ovules and forms ectopic carpel structures (Ragni et al. 2008). And in our previous study, we demonstrated that *AG* may be involved in the formation of Japanese apricot multi-pistil, so the gene function of *PmKNAT2* in *P. mume* is well worth studying in depth (Shi et al. 2023). To further analyze the role of *PmKNAT2/6-a* and *PmKNAT2/6-b* in pistil number determination in Japanese apricot, reverse transcription quantitative PCR (RT-qPCR) experiments were conducted on flower buds from the pistil differentiation stage of 2 pistil varieties, ‘Longyan’ (‘LY’, single-pistil variety) and ‘Dayu’ (‘DY’, multi-pistil variety) (Fig. 1B). The analysis revealed that *PmKNAT2/6-a* expression was significantly higher in ‘DY’ than in ‘LY’, indicating that this gene may play an active role in the development of multiple pistils in Japanese apricot. There was no significant difference between ‘LY’ and ‘DY’ in the expression of *PmKNAT2/6-b*, suggesting that this gene, although involved in pistil morphogenesis, may not be a key gene for the development of multiple pistils. The CDS regions of *PmKNAT2/6-a* and *PmKNAT2/6-b* in ‘LY’ and ‘DY’ were cloned to investigate the function of the genes, and the amino acid sequences of PmKNAT2/6-a (GeneID_103343109) and PmKNAT2/6-b (GeneID_103321797) of these 2 species were identical. DNA sequence analysis (exon, intron, and promoter analysis) showed no substantial differences or polymorphisms between the 2 cultivars (Supplementary Figs. S1 and S2). The phenotypes of Arabidopsis lines overexpressing *PmKNAT2/6-a* (OE-PmKNAT2/6-a) and *PmKNAT2/6-b* (OE-PmKNAT2/6-b) compared to the wild type (WT) revealed that OE-PmKNAT2/6-a could induce a multi-pistillate phenotype, whereas OE-PmKNAT2/6-b could not (Fig. 1, C and D; Supplementary Figs. S3 and S4). The increased number of pistils in OE-PmKNAT2/6-a flowers was accompanied by abnormal development and an increase in the number of other floral organs, resulting in these flowers being unable to produce seeds. Although *PmKNAT2/6-b* may not be involved in multi-pistil formation, the curved pistils of OE-PmKNAT2/6-b indicate that *PmKNAT2/6-b* is also involved in flower development.

### PmAGL24 directly binds to the *PmKNAT2/6-a* promoter

**Figure 2.**
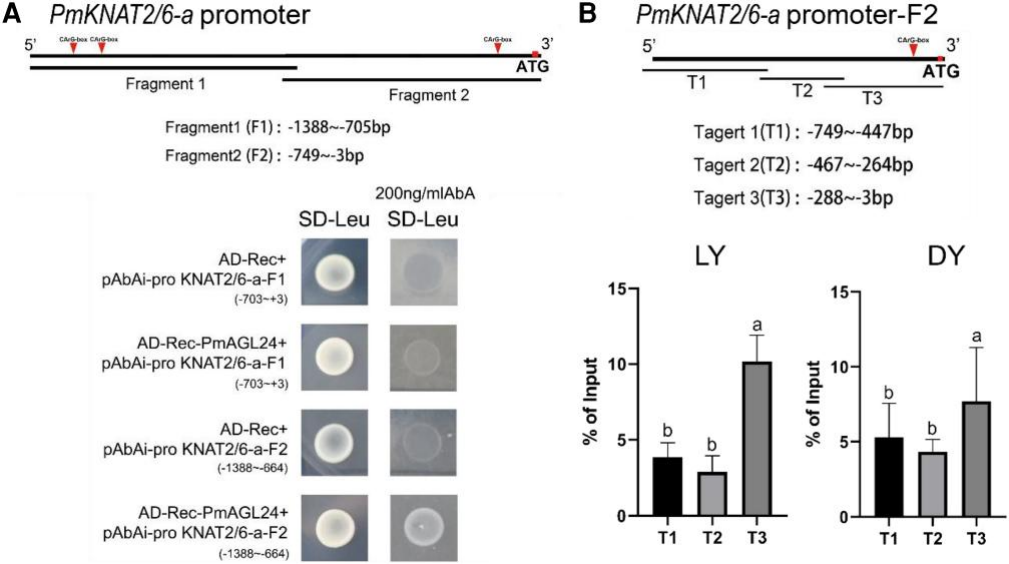
PmAGL24 is a key transcriptional regulator upstream of *PmKNAT2/6-a* in Japanese apricot. **A)** Physical interaction of PmAGL24 with the *PmKNAT2/6-a* promoter in a yeast one-hybrid (Y1H) assay. The promoter of *PmKNAT2/6-a* was divided into Fragments 1 (F1) and 2 (F2), and yeast co-transformed with different constructs was grown on SD/-Leu medium with or without AbA. The horizontal lines represent the fragment range. AD-Rec-PmAGL24 was used as the prey, pAbAi-proKNAT2/6-a-F1 and pAbAi-proKNAT2/6-a-F2 as the baits, and AD-Rec as the control. **B)** ChIP-qPCR evidence of PmAGL24 binding to the *PmKNAT2/6-a* promoter in vivo. The PmKNAT2/6-a-F2 region promoter was divided into Targets 1 (T1), 2 (T2), and 3 (T3). Horizontal lines represent intervals of the ChIP-qPCR primer design. PmAGL24 binds specifically to the T3 region of the *PmKNAT2/6-a* promoter in the 2 Japanese apricot cultivars ‘LY’ and ‘DY’ according to ChIP-qPCR assays. Values are the percentage of DNA samples immunoprecipitated with the anti-PmAGL24 antibody relative to the input DNA. Error bars represent Se based on 3 biological replicates. Letters indicate significant differences using Student's *t*-test (*P* < 0.05).

To investigate the upstream regulatory mechanism of the *PmKNAT2/6-a* gene associated with Japanese apricot pistil differentiation, we identified the *Prunus mume* AGAMOUS-like 24 (PmAGL24) transcription factor that regulates *PmKNAT2/6-a* by a yeast one-hybrid (Y1H) screening library (Supplementary Figs. S5 to S9; Supplementary Table S1). PmAGL24 is a member of the *MADS-box* family, and all members of this gene family can recognize the CArG-box (CC[A/T]_6_GG or CC[A/T]_7_GG). To study the binding region of the *PmKNAT2/6-a* promoter by PmAGL24, the sequence of the *PmKNAT2/6-a* promoter was analyzed, and the promoter region contained 3 CArG-boxes. The *PmKNAT2/6-a* promoter was divided into Fragments 1 (F1, containing 2 CArG-box) and 2 (F2, containing 1 CArG-box) (Fig. 2A; Supplementary Fig. S10; Supplementary Table S2). First, comparing the PmAGL24 sequences obtained by cloning from ‘LY’ and ‘DY’ revealed that neither sequence contained a terminator, frameshift or key functional, structural domain mutation. Because the difference in gene sequence between these 2 Japanese apricot varieties was not the primary cause of differences in the number of pistils in Japanese apricot, subsequent experiments used *PmAGL24* from ‘LY’ as the source material (GeneID_103319497) (Supplementary Table S3). PmAGL24 could bind to the CArG box site of the *PmKNAT2/6-a-*F2, which is a region of the *PmKNAT2/6-a* promoter capable of binding.

We designed peptide antibodies against PmAGL24 and conducted chromatin immunoprecipitation-quantitative polymerase chain reaction (ChIP-qPCR) experiments with flower buds of 2 varieties of Japanese apricot, ‘LY’ and ‘DY’, at the critical time point for determining the pistil number. The *PmKNAT2/6-a*-F2 promoter region was divided into 3 sections: Targets 1 (T1), 2 (T2), and 3 (T3, which contained the binding site CArG-box) (Fig. 2B). The 3 fragments were measured using the enriched DNA solution as a template, and the experiments were conducted according to the kit's instructions (ChIP Kit-Plants-ab117137). PmAGL24 could bind to the promoter of *PmKNAT2/6-a* in Japanese apricot, with the CArG-box in the T3 region acting as the binding site.

### PmAGL24 represses *PmKNAT2/6-a* expression at the transcriptional level

**Figure 3.**
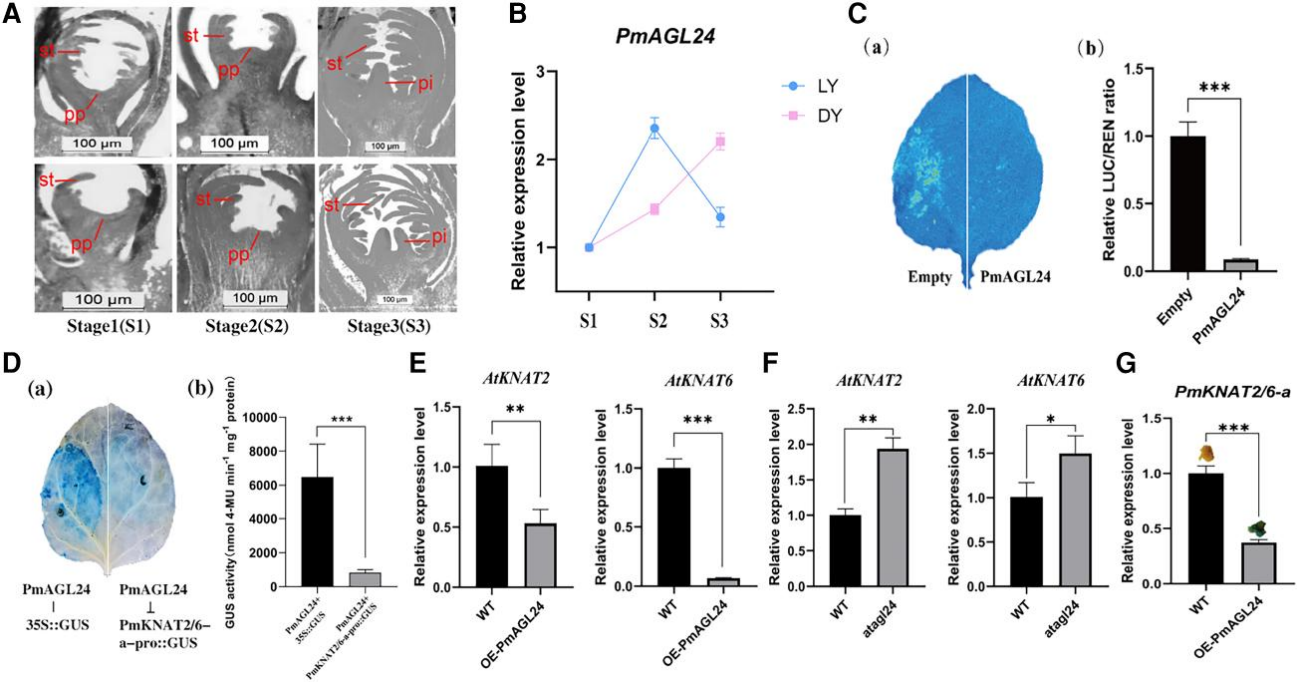
PmAGL24 is involved in determining pistil number in Japanese apricot. **A)** The paraffin sections show the process of pistil number differentiation in Japanese apricot. The upper row shows a paraffin section of flower buds from ‘LY’; the lower row shows a paraffin section of flower buds from ‘DY’. Stage 1 represents undifferentiated, Stage 2 represents early differentiation, and Stage 3 represents differentiated. st, stamen; pp, pistil primordium; pi, pistil. **B)** Expression analysis of *PmAGL24* in ‘LY’ and ‘DY’. **C)** Dual-luciferase assay showing that PmAGL24 repressed *PmKNAT2/6-a* promoter activity. The LUC/REN group with empty vector + p2301-*PmAGL24* is shown on the left, and the pGreen II 0800-LUC*-PmKNAT2/6-a*-pro + p2301-*PmAGL24* group is shown on the right. (a) is luciferase complementation imaging and (b) is relative LUC/REN ratio. Images were digitally extracted for comparison. Error bars represent Se based on 3 biological replicates. Asterisks indicate significant differences using Student's *t*-test (***P < 0.001; **P < 0.01; *P < 0.05). **D)** GUS immunohistochemistry verified that PmAGL24 inhibited the activity of *PmKNAT2/6-a* promoter. 35S::GUS on the left and PmKNAT2/6-a-pro::GUS on the right. (a) is GUS immunohistochemistry and (b) is GUS activity. Images were digitally extracted for comparison. Error bars represent Se based on 3 biological replicates. Asterisks indicate significant differences using Student's *t-*test (***P < 0.001; **P < 0.01; *P < 0.05). **E and F)** Expression analysis of *AtKNAT2* and *AtKNAT6* in transgenic Arabidopsis. Error bars represent Se based on 3 biological replicates. Asterisks indicate significant differences using Student's *t-*test (***P < 0.001; **P < 0.01; *P < 0.05). D is expression analysis of *AtKNAT2* and *AtKNAT6* in overexpression *PmAGL24* Arabidopsis. E is expression analysis of *AtKNAT2* and *AtKNAT6* in *atagl24* mutant Arabidopsis. WT represents the wild type. **G)** Expression analysis of *PmKNAT2/6-a* in Japanese apricot callus overexpressing *PmAGL24*. Error bars represent Se based on 3 biological replicates. Asterisks indicate significant differences using Student's *t*-test (***P < 0.001; **P < 0.01; *P < 0.05). The top of each bar is the corresponding GUS-stained callus tissue. WT represents the wild type.

*PmAGL24*, also known as *Prunus mume dormancy-associated MADS6* (*PmDAM6*), is currently the main research direction for promoting flowering and dormancy release (Liu et al. 2009, Kitamura et al. 2016). In Arabidopsis, the *agl24 svp* double mutant severely impairs pistil development at 30 °C, suggesting that *AGL24* may be involved in the pistil morphogenesis process (Gregis et al. 2006). However, whether *AGL24* is involved in forming multiple carpels has not been reported and deserves further investigation. The early stage of pistil differentiation in Japanese apricot is divided into 3 main periods: undifferentiated (Stage 1), early differentiated (Stage 2), and differentiated (Stage 3) (Fig. 3A), with the period between Stages 1 and 2 being the decisive period for pistil number differentiation. In ‘LY’ and ‘DY’, RT-qPCR experiments were carried out with flower buds from these 3 periods (Fig. 3B). *PmAGL24* expression in ‘LY’ abruptly increased to a peak during Stages 1 and 2, then fell off during Stages 2 and 3. In contrast, *PmAGL24* expression in ‘DY’ increased gradually at each stage. The discovery that *PmAGL24* expression was higher in the single-pistil Japanese apricot cultivar ‘LY’ at the critical stage of pistil differentiation is consistent with the experimental results in the previous chapter that *PmAGL24* suppresses *PmKNAT2/6-a* expression. A transient dual-luciferase-based transactivation assay was used to determine whether PmAGL24 could activate the transcription of *PmKNAT2/6-a* (Supplementary Fig. S11). *Nicotiana benthamiana* leaves containing the firefly luciferase reporter gene (LUC) were co-injected with a construct overexpressing PmAGL24 as an effector. Figure 3C illustrates that PmAGL24 decreased the activity of the *PmKNAT2/6-a* promoter, resulting in a significantly lower LUC/Renilla luciferase (REN) ratio compared to the control. To further investigate, we added a more detailed study to investigate the regulatory mechanism of PmAGL24 on *PmKNAT2/6* by GUS staining experiments. The results of the study were consistent with the previous findings that PmAGL24 was able to inhibit the expression of the *PmKNAT2/6* gene (Fig. 3D; Supplementary Fig. S12).

In addition, genetic transformation tests for *PmAGL24* in Arabidopsis were carried out (Supplementary Fig. S13A). We found that *PmAGL24* heterologous expression, although not directly affecting changes in pistil number in Arabidopsis (Supplementary Fig. S14), was able to suppress the expression of *AtKNAT2* and *AtKNAT6* in Arabidopsis (Fig. 3E). Meanwhile, CRISPR-Cas9 knockdown of *Atagl24* in Arabidopsis led to a significant increase in the expression of *AtKNAT2* and *AtKNAT6* (Fig. 3F; Supplementary Figs. S13B and S14). Genetic transformation studies of *PmAGL24* on Japanese apricot callus supported our findings by demonstrating that *PmAGL24* overexpression in Japanese apricot directly resulted in a significant reduction in *PmKNAT2/6-a* expression (Fig. 3G).

**Figure 4.**
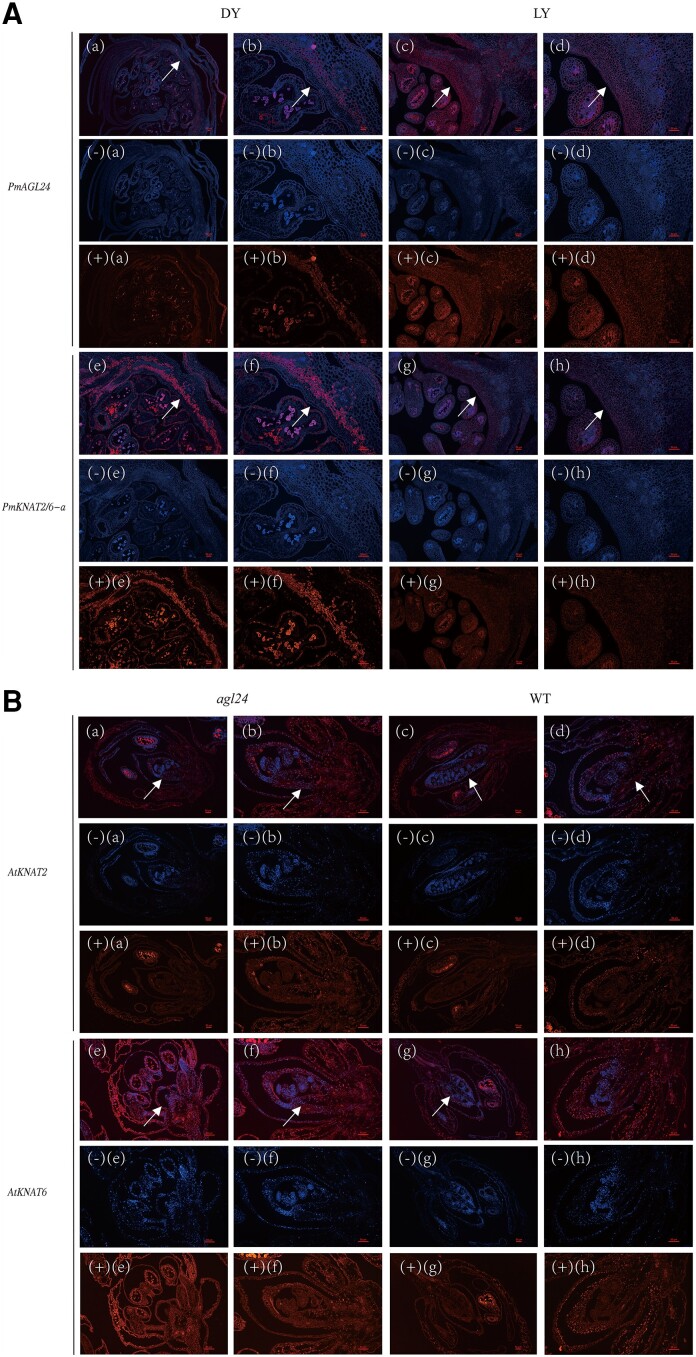
FISH analysis of *PmAGL24*, *PmKNAT2/6-a*, *AtKNAT2*, and *AtKNAT6*. **A)** FISH analysis of *PmAGL24* and *PmKNAT2/6-a* in ‘DY’ and ‘LY’. (a) to (d) are pictures of the merging of red fluorescent protein (RFP) of PmAGL24 and 4′,6-diamidino-2-phenylindole (DAPI) signals. (−a) to (−d) are DAPI (blue) signals. (+a) to (+d) are PmAGL24 (red) signals. (b) and (d) an amplification of a region marked by a white arrow in (a) and (c), respectively. (e) to (h) are pictures of the merging of red fluorescent protein (RFP) of PmKNAT2/6-a and DAPI signals. (−e) to (−h) are DAPI (blue) signals. (+e) to (+h) are PmKNAT2/6-a (red) signals. (f) and (h) an amplification of a region marked by a white arrow in (e) and (g), respectively. The first 2 vertical lines are ‘DY’, the last 2 vertical lines are ‘LY’. **B)** FISH analysis of *AtKNAT2* and *AtKNAT6* in *atagl24* mutant and wild-type Arabidopsis. (a) to (d) are pictures of the merging of red fluorescent protein (RFP) of *AtKNAT2* and DAPI signals. (−a) to (−d) are DAPI (blue) signals. (+a) to (+d) are *AtKNAT2* (red) signals. (b) and (d) an amplification of a region marked by a white arrow in (a) and (c), respectively. (e) to (h) are pictures of the merging of red fluorescent protein (RFP) of *AtKNAT6* and DAPI signals. (−e) to (−h) are DAPI (blue) signals. (+e) to (+h) are *AtKNAT6* (red) signals. (f) and (h) an amplification of a region marked by a white arrow in (e) and (g), respectively. The first 2 vertical lines are *atagl24* mutant, the last 2 vertical lines are wild-type Arabidopsis. The scale bar represents 50 *μ*m.

The critical period for the differentiation of pistil number is before Stage2 at the early stage of pistil differentiation in Japanese apricot (Fig. 3A). To assess the expression patterns of *PmAGL24* and *PmKNAT2/6-a* during the development of the pistil primordium, their expression was detected using fluorescence in situ hybridization (FISH) (Fig. 4A). The right image in each set of photographs is an enlarged view of the position indicated by the white arrow in the left image. It can be observed from the figure that the expression of *PmAGL24* was substantially lower in ‘DY’ than in ‘LY’, while the expression of *PmKNAT2/6-a* showed the opposite trend. This is consistent with the previous findings. To further investigate whether there is a direct inhibitory effect of AGL24 on *KNAT2/6*, we analyzed further FISH experiments using the *agl24* mutant and wild-type Arabidopsis (Fig. 4B). Loss of function of *agl24* was found to lead to an increase in *KNAT2/6* gene expression both when the pistil is just forming and when the pistil morphology is already formed in Arabidopsis. The above results support the conclusion that PmAGL24 represses *PmKNAT2/6-a* at the transcriptional level.

### PmLHP1 recognizes H3K27me3 and binds *PmKNAT2/6-a* to participate in Japanese apricot pistil determination

Previous studies in this paper demonstrated that PmAGL24 is involved in pistil number differentiation in Japanese apricot and can act as a transcription factor binding to the *PmKNAT2/6-a* promoter to repress the expression of this gene. However, heterologously expressing the *PmAGL24* gene in Arabidopsis, no change in carpel number was observed. There are many reasons for this situation, such as that the gene function of PmAGL24 is supposed to prevent excessive differentiation of pistillate primordia during the quantitative differentiation phase and that the gene function of *PmAGL24* is redundant during the pistillate differentiation phase. However, such results also led us to wonder whether there are other mechanisms involved in the repressive effect of PmAGL24 on the *PmKNAT2/6-a* gene, similar to double insurance, to ensure the normal differentiation of the pistil number.

**Figure 5.**
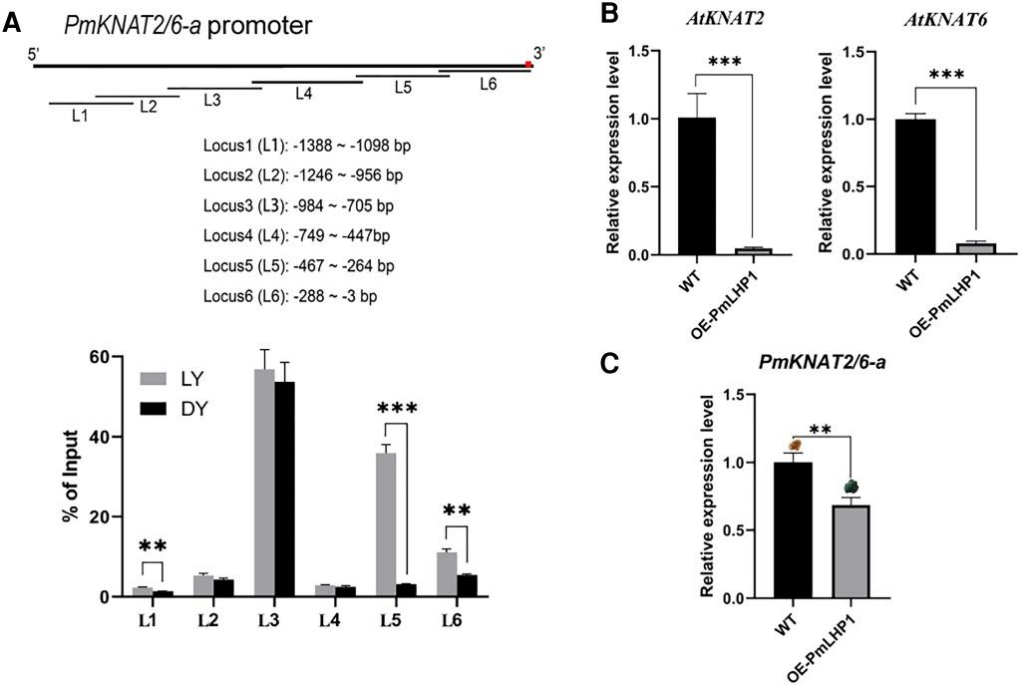
PmLHP1 recruits H3K27me3 to regulate *KNAT2/6-a* gene expression in Japanese apricot. **A)** ChIP-qPCR was used to detect H3K27me3 modification on the *PmKNAT2/6-a* gene locus. The primer design position diagram is shown above. The horizontal line represents the primer design interval for ChIP-qPCR. L1 represents Locus 1, L2 represents Locus 2, and so on. The bottom is the site-specific analysis of H3K27me3 binding to the *PmKNAT2/6-a* promoter in ‘LY’ and ‘DY’. Asterisks indicate significant differences using Student's *t*-test (***P < 0.001; **P < 0.01). **B)** Expression analysis of *AtKNAT2* and *AtkKNAT6* in Arabidopsis heterologously expressing *PmLHP1*. Error bars represent Se based on 3 biological replicates. Asterisks indicate significant differences using Student's *t*-test (***P < 0.001). **C)** Expression analysis of *PmKNAT2/6-a* in Japanese apricot callus overexpressing *PmLHP1*. Error bars represent Se based on 3 biological replicates. Asterisks indicate significant differences using Student's *t*-test (**P < 0.01). The top of each bar is the corresponding GUS-stained callus tissue. WT represents the wild type.

In a previous study, we identified the epigenetic factor PmLHP1 with the function of recognizing and recruiting H3K27me3 and its important role in the pistil number differentiation process in Japanese apricot (Shi et al. 2023). Since the Class I *KNOTTED1-like homeobox* (*KNOX*) genes in Arabidopsis were enriched in tri-methylated lysine 27 of histone H3 (H3K27me3) modifications (Zhang et al. 2007), we hypothesized that histone methylation is involved in the PmKNAT2/6-a-mediated multi-pistil construction process. To determine whether histone modifications are involved in PmKNAT2/6-a-mediated pistillogenesis in Japanese apricot, we purchased commercially available anti-H3K27me3 antibodies and conducted ChIP-qPCR experiments on flower buds from 2 Japanese apricot cultivars ‘LY’ and ‘DY’. The promoter region of *PmKNAT2/6-a* was enriched at the H3K27me3 modification site, with higher enrichment efficiency in the Locus3, Locus5, and Locus6 regions (Fig. 5A). The H3K27me3 level of ‘LY’ was significantly higher than that of ‘DY’ in Locus1, Locus5, and Locus6 regions, where the Locus6 region overlapped with the position of PmAGL24 directly binding to the *PmKNAT2/6-a* promoter region. This suggests that H3K27me3 modification may be involved in the PmKNAT2/6-a-related multi-pistil construction process.

In a previous study, we found that PmLHP1 plays a key role in regulating floral number differentiation and that the Arabidopsis double mutant *ag-11 lhp1* exhibits a multi-pistil phenotype. Heterologous expression of *PmLHP1* rescued this phenotype, resulting in a normal single-pistil phenotype (Shi et al. 2023). To determine whether PmLHP1 is involved in the H3K27me3 histone modification process of *PmKNAT2/6-a*, RT-qPCR experiments were conducted using Arabidopsis heterologously expressing *PmLHP1* (Supplementary Figs. S15 to S17). The results showed that the heterologous expression of *PmLHP1* significantly decreased the expression of *AtKNAT2* and *AtKNAT6* in Arabidopsis (Fig. 5B). In Japanese apricot callus overexpressing *PmLHP1*, *PmKNAT2/6-a* expression was significantly suppressed (Fig. 5C). The above results suggest that PmLHP1 plays a role in multi-pistil formation in Japanese apricot by maintaining H3K27me3 in a manner that inhibits *PmKNAT2/6-a*.

### PmAGL24 can recruit PmLHP1 to participate in pistil determination by inhibiting *PmKNAT2/6-a* via H3K27me3

**Figure 6.**
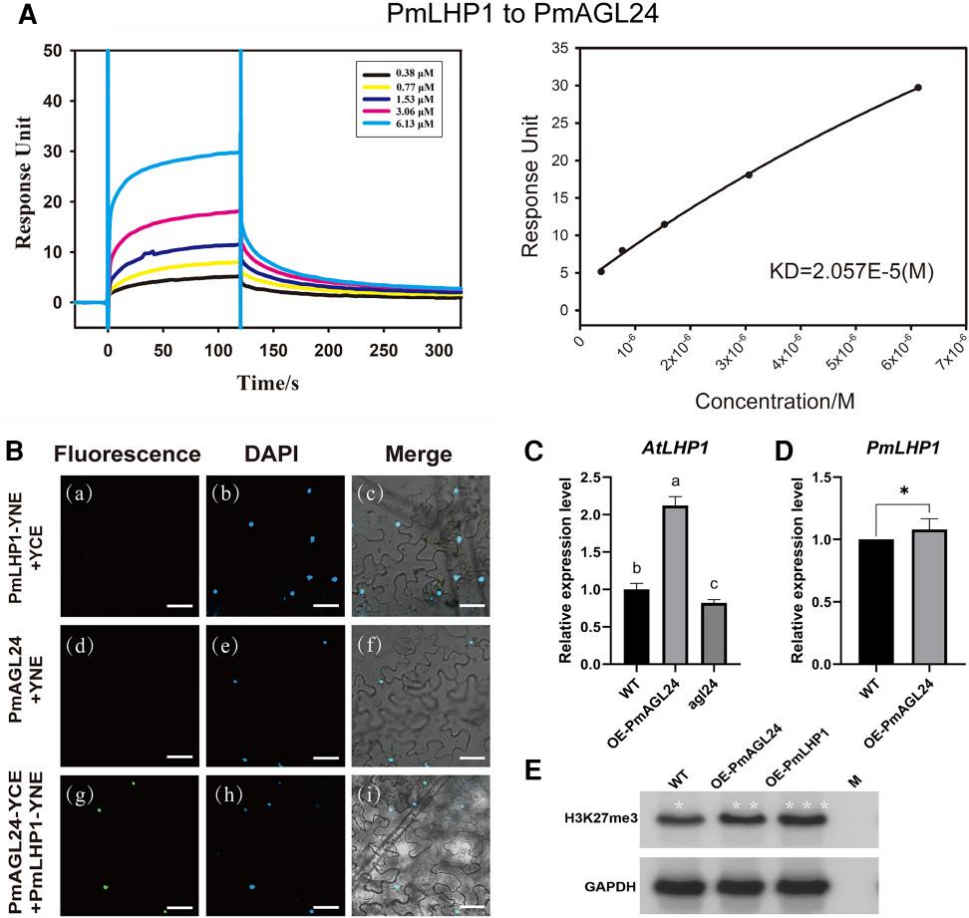
Protein interactions exist between PmAGL24 and PmLHP1 in vitro and in vivo. **A)** SPR to verify the existence of PmAGL24 and PmLHP1 interactions in vitro. Equilibrium binding curves and derived dissociation constants for each pair of interacting proteins are shown. The KD values are expressed as the mean ± Sd, *n* = 2. SPR sensor plots are provided as source data files. **B)** BiFC assay to verify the existence of interactions between PmAGL24 and PmLHP1 in vivo. All images were taken by confocal microscopy (ZEISS, LSM800). PmAGL24-YCE + PmLHP1-YNE was the experimental group. PmLHP1-YNE + YCE (a to c) and PmAGL24-YCE + YNE (d to f) were used as negative controls. The white bar represents 10 *μ*m. **C)** Expression analysis of *AtLHP1* in heterologous expression of *PmAGL24* in Arabidopsis. Error bars represent Se based on 3 biological replicates. Letters indicate significant differences using Student's *t*-test (*P* < 0.05). **D)** Expression analysis of *PmLHP1* in Japanese apricot callus overexpressing *PmAGL24*. The top of each bar is the corresponding GUS-stained callus tissue. Error bars represent Se based on 3 biological replicates. Asterisks indicate significant differences using Student's *t*-test (*P < 0.05). WT represents the wild type. **E)** H3K27me3 modification in Japanese apricot callus overexpressing *PmAGL24* and *PmLHP1*. The white asterisk above the bands are not statistically significant, just represent the gray value; the more asterisks, the greater the gray value (Supplementary Table S4). GAPDH is the positive control for the quantification of tissue samples. M represents the marker. WT represents the wild type.

It is now evident that PmLHP1 can inhibit the role of *PmKNAT2/6-a* in pistil number differentiation in Japanese apricot by maintaining H3K27me3, but it is still unknown how PmLHP1, as a protein, is recruited to the promoter of *PmKNAT2/6-a*. Previous research has shown that PmAGL24 recognizes and binds to the CArG-box near the 5′ end of the *PmKNAT2/6-a* promoter, and the region where this binding domain is located overlaps with the H3K27me3 modification-enriched region. Thus, it is speculated that PmAGL24 regulates pistil number differentiation in Japanese apricot by recruiting PmLHP1 to the promoter of *PmKNAT2/6-a* through protein interactions to establish H3K27me3 and repress *PmKNAT2/6-a* expression. We conducted surface plasmon resonance (SPR) experiments using recombinant His-LHP1 and Gst-AGL24 proteins that were purified (Supplementary Fig. S18). Our SPR results showed a direct interaction between the proteins of PmAGL24 and PmLHP1, with a binding activity of 2.057 × 10^−5^ M (Fig. 6A). To validate the SPR protein interaction results, we performed bimolecular fluorescence complementation (BiFC) and co-transformed *PmAGL241* and *PmLHP1* into *N. benthamiana* epidermal cells (Supplementary Fig. S19). The results revealed a direct protein interaction between PmAGL24 and PmLHP1 in plants (Fig. 6B), consistent with the SPR results. We focused on the *AtLHP1* gene's expression in Arabidopsis by overexpressing *PmAGL24* and knocking down *atagl24*, and we discovered that while heterologous expression of *PmAGL24* increased *AtLHP1* expression, knocking down *AGL24* decreased *AtLHP1* expression (Fig. 6C). Homologous *PmAGL24* expression (Fig. 6D) also showed a significant increase in *PmLHP1* expression, indicating that PmAGL24 may be able to recruit PmLHP1. We carried out western blot experiments to further highlight PmAGL24's impact on H3K27me3 modification. Although it was not as high as it was in overexpressed *PmLHP1* (Fig. 6E), 35S:*PmAGL24* substantially increased H3K27me3 modification in Japanese apricot (Supplementary Table S4). These results suggest that PmAGL24 could recruit PmLHP1 to establish H3K27me3 in Japanese apricot.

## Discussion

In the ABCDE regulatory model of Arabidopsis flower development, the class C gene *AG* is involved in pistil determination and development, and it can terminate stem cell maintenance by repressing *WUSCHEL* (*WUS*) expression directly or indirectly via *KNUCKLES* (*KNU*) (Bowman et al. 1991, Schneitz 1999, Lenhard et al. 2001). *KNOTTED1-like homeobox2* (*KNAT2*) ectopic expression induces the production of carpel-like bead structures (Ragni et al. 2008), thereby expanding the range of *AG* gene expression in carpels and ovules; however, this induction of carpel structures is independent of the effects of *AG* genes (Pautot et al. 2001). The ASSYMETRICLEAVES1 (AS1) transcription factor with the MYB structural domain can form a heterodimer AS1/AS2 with the ASYMMETRIC LEAVES2 (AS2) protein with the LATERAL ORGAN BOUNDARIES (LOB) structural domain in Arabidopsis, and AS1/AS2 binds directly to the promoter of the *KNAT2* gene. AS1/AS2 binds directly to the *KNAT2* promoter region and negatively regulates *KNAT2* expression. *KNAT2* is regulated by CTK, gibberellin, and growth hormone. It regulates the expression of some CTK synthase genes, promotes CTK accumulation, and activates the CTK response (Hay and Tsiantis 2010). The CTK content determination we performed showed that the amount of CTK in ‘DY’ was significantly higher than that in ‘LY’ validation (Supplementary Table S5).

The results of this study shed light on the regulatory mechanisms involved in the development of pistil number differentiation in Japanese apricot. Heterologous expression of the *PmKNAT2/6-a* gene in Arabidopsis resulted in a multi-pistil phenotype, indicating its importance in the development of multiple pistils in Japanese apricot. The transcription factor PmAGL24, known to regulate flowering and dormancy release, was identified as an upstream regulator of *PmKNAT2/6-a*. PmAGL24 directly bound to the promoter and suppressed *PmKNAT2/6-a* expression. Interestingly, *PmAGL24* overexpression did not cause a change in the carpel number in Arabidopsis, suggesting potential functional redundancy with other genes involved in carpel development. Further investigation revealed that the *atagl24* mutant in Arabidopsis displayed increased expression of *AtKNAT2* and *AtKNAT6*, homologs of *PmKNAT2/6-a*, indicating a repressive effect of AGL24 on the expression of *KNAT2* genes. Epigenetic mechanisms play a role in the regulation of pistil number differentiation. The enrichment of H3K27me3 modification, a crucial epigenetic marker, was observed on the motif of *PmKNAT2/6-a*, similar to observations in Arabidopsis. The protein PmLHP1, known to recognize and recruit H3K27me3, repressed several genes involved in pistil number development in Japanese apricot. The expression levels of PmLHP1 and H3K27me3 were higher during the critical period of pistil number differentiation in single-pistil Japanese apricot varieties, suggesting their involvement in pistil number determination. *PmLHP1* was shown to repress *PmKNAT2/6-a* expression, potentially by recruiting H3K27me3. In our experiments, we examined the expression levels of the *AtKNAT2* and *AtKNAT6* genes using Arabidopsis *lhp1* mutants and wild-type plants. Our findings revealed that the expression of *AtKNAT2* did not exhibit significant changes when the *AtLHP1* gene was mutated. However, there was a highly significant decrease in the expression of the *AtKNAT6* gene in the *lhp1* mutants compared to wild-type plants (Supplementary Fig. S20). These results suggest that a complex regulatory relationship may exist between the expression of *AtLHP1* and *AtKNAT6* in Arabidopsis.

Previous studies have shown that class I *KNOX* genes in Arabidopsis are crucial for maintaining the undifferentiated state of stem cells in meristematic tissues (Lincoln et al. 1994). Regulatory mechanisms, including the AS1/AS2 complex and the AS2/TCP complex, have been identified as repressing class I *KNOX* genes (Guo et al. 2008, Li et al. 2012b). In our study, we identified a transcription factor, PmAGL24, upstream of *PmKNAT2/6-a*, which is known to regulate flowering and dormancy release (Yu et al. 2004, Kitamura et al. 2016). By screening the Y1H screen library, we found that PmAGL24 may directly bind to the promoter and suppress *PmKNAT2/6-a* expression. However, when we heterologously expressed the *PmAGL24* gene in Arabidopsis, we did not observe any changes in the carpel number. This could be attributed to multiple factors, such as the role of the *PmAGL24* gene in preventing excessive differentiation of pistillate primordia during the quantitative differentiation phase or the presence of redundant genes during pistillate differentiation. In Arabidopsis, the *atagl24* mutant also did not cause any phenotypic changes, and it is speculated that other genes (e.g. *SVP* and *FLC*) may function in the atagl24 mutant to compensate for the lack of function of AGL24 in combination with previous work (Gregis et al. 2009). Further supporting this, the *atagl24* mutant showed increased expression levels of *AtKNAT2* and *AtKNAT6*, homologs of *PmKNAT2/6-a*, indicating a repressive effect of AGL24 on the expression of *KNAT2* genes.

To provide direct evidence of the transcriptional repressive effect of PmAGL24 on *PmKNAT2/6-a*, we examined *PmKNAT2/6-a* expression in Japanese apricot callus overexpressing *PmAGL24*. We observed a significant decrease in *PmKNAT2/6-a* expression, confirming the repressive effect of PmAGL24. Additionally, previous studies have shown that heterologous expression of *PmAGL24* reduces the CTK content (Yamane et al. 2019), and in Arabidopsis, KNAT2 expression is positively associated with CTK levels (Jasinski et al. 2005), suggesting a negative regulatory relationship between *PmAGL24* and the *PmKNAT2* gene. Further research is needed to explore other regulatory modalities involved in the repression of *PmKNAT2/6-a* by PmAGL24.

The emergence of epigenetics has revolutionized our understanding of regulatory pathways and metabolic pathways, providing perspectives and methodologies for studying plant growth and development mechanisms. One important epigenetic modification is the regulation of H3K27me3, which acts as a switch to stabilize expression changes in hundreds of transcription factors, subsequently influencing the transcriptional changes of thousands of genes (Müller-Xing et al. 2022). To further investigate the upstream regulatory mechanism of *PmKNAT2/6-a*, we analyzed the distribution of H3K27me3 on the gene motif. Our analysis revealed that the motif of *PmKNAT2/6-a* was enriched at H3K27me3 modification sites, similar to observations in Arabidopsis (Zhang et al. 2007). PmLHP1 specifically recognizes and recruits H3K27me3 and represses a variety of genes involved in pistil number development (Shi et al. 2023). Western blot assays demonstrated higher expression levels of PmLHP1 and H3K27me3 during the critical period of pistil number differentiation in single-pistil Japanese apricot varieties, and their expression trends showed a positive association, suggesting the potential joint involvement of PmLHP1 and H3K27me3 in pistil number differentiation in Japanese apricot. Further investigations confirmed that PmLHP1 can indeed recognize H3K27me3 in Japanese apricot. However, in Arabidopsis flowers heterologously expressing *PmLHP1*, there was no substantial difference in the number of carpels compared to the wild type (Supplementary Fig. S17). This finding is consistent with observations in Arabidopsis lhp1-2 mutants, which exhibit a multi-pistil phenotype (Liu et al. 2014), indicating that LHP1 deletion affects pistil number differentiation, but there is functional redundancy in excess LHP1. Analysis of gene expression showed that *PmLHP1* strongly repressed the *PmKNAT2/6-a* gene, and it was hypothesized that *PmLHP1* represses *PmKNAT2/6-a* gene expression by recruiting H3K27me3.

Our study identified that the modification site of H3K27me3 overlaps with the PmAGL24-binding site of the *PmKNAT2/6-a* promoter and that PmLHP1 recognizes H3K27me3, leading to the speculation that PmAGL24 may be linked to the establishment of H3K27me3 modification on the *PmKNAT2/6-a* promoter. The study demonstrated the existence of protein interactions between PmAGL24 and PmLHP1. When PmAGL24 was overexpressed in Japanese apricot callus, the expression level of PmLHP1 increased significantly, indicating that PmAGL24 has the function of recruiting PmLHP1 gene expression. Western Blot (WB) assays revealed that H3K27me3 modification was significantly increased in Japanese apricot callus overexpressing PmAGL24 and PmLHP1, indicating that PmAGL24 and PmLHP1 could recruit H3K27me3 to establish modifications at the protein level. In conclusion, the above study revealed that PmAGL24 in Japanese apricot can directly suppress the expression level of *PmKNAT2/6-a* by binding to the promoter and indirectly suppress the expression level of *PmKNAT2/6-a* by forming a protein complex with PmLHP1 to recruit H3K27me3 at the *PmKNAT2/6-a* promoter to ensure normal differentiation of the number of pistils. Such a mechanism has not been reported in other species and may be unique to Japanese apricot or Rosaceae, although further study is needed to verify this.

**Figure 7.**
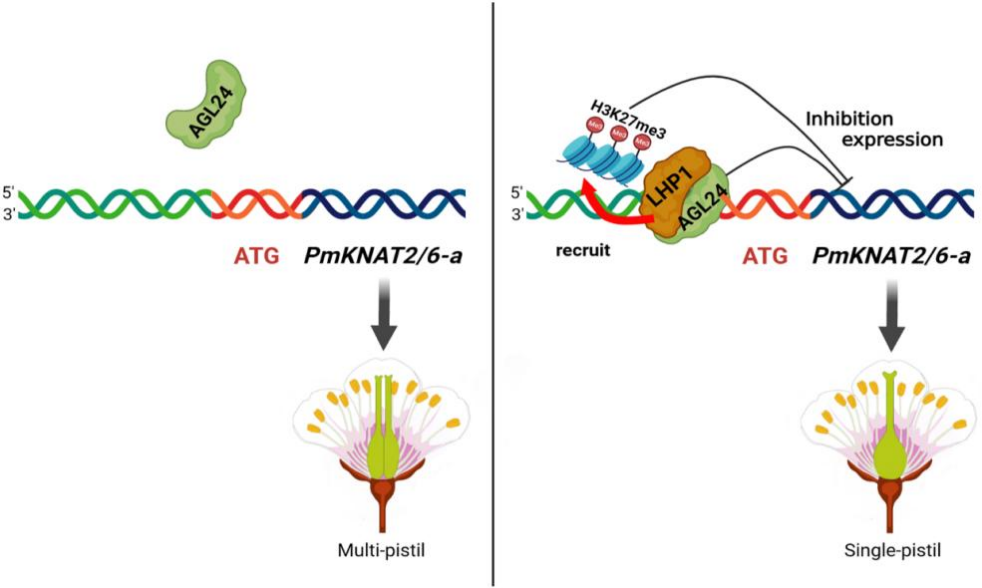
Schematic representation of PmAGL24 regulating *PmKNAT2/6-a* gene expression directly and indirectly. On the left, the gradual increase in expression of *PmAGL24* leads to uncontrolled expression of the *PmKNAT2/6-a* gene, resulting in the formation of multi-pistil Japanese apricot. On the right, PmAGL24 binds to the *PmKNAT2/6-a* promoter to repress gene expression while also binding to PmLHP1 via a protein–protein interaction, which recruits H3K27me3 to the *PmKNAT2/6-a* promoter, repressing transcriptional activity and resulting in the Japanese apricot single-pistil phenotype.

Based on our findings, we have proposed 2 models to elucidate the regulatory role of PmAGL24 in maintaining normal pistil differentiation in Japanese apricot. These models provide insights into how PmAGL24 regulates the expression of the *PmKNAT2/6-a* gene, either directly or indirectly. In the first model (Fig. 7, left), during the early stages of pistil number differentiation, PmAGL24 exhibited low expression levels and displayed weak or no binding to the promoter region of the *PmKNAT2/6-a* gene. Consequently, *PmKNAT2/6-a* expression remained unrestricted, leading to a multi-pistil phenotype in Japanese apricot. In contrast, the second model (Fig. 7, right) proposed that at the initial stage of pistil differentiation, *PmAGL24* was highly expressed. In this scenario, PmAGL24 exerted regulatory control by directly binding to the promoter of the *PmKNAT2/6-a* gene, resulting in the repression of its expression. Furthermore, through its interaction with PmLHP1, PmAGL24 formed a protein complex that recruited H3K27me3 to establish repressive modifications at the *PmKNAT2/6-a* promoter. This dual repression mechanism involving both direct binding and recruitment of H3K27me3 led to the mono-pistil phenotype observed in Japanese apricot.

These findings highlight the necessity of distinct transcriptional and regulatory mechanisms acting simultaneously on the *PmKNAT2/6-a* gene to ensure proper pistil number differentiation. The proposed models contribute to our understanding of how PmAGL24 governs this process and emphasize the intricate nature of the regulatory networks involved in pistil development.

## Materials and methods

### Plant material collection and growth conditions

The floral buds of Japanese apricot (*P. mume* Sieb. et Zucc.) cv ‘Longyan’ (‘LY’, single-pistil variety) and ‘Dayu’ (‘DY’, multi-pistil variety) used in this study were collected at the National Field GenBank for *P. mume* in Nanjing, Jiangsu Province, China. All samples were immediately frozen in liquid nitrogen and stored at −80 °C until further analysis.

### Constructing the phylogenetic tree

The *Prunus mume* genome (GCF_000346735.1 *P.mume*_V1.0) file and its annotation files were downloaded from NCBI (https://www.ncbi.nlm.nih.gov/). Hidden Markov models (pfam03790, pfam03791, pfam03789, and pfam05920) containing PFAM (http://pfam.xfam.org/) were retrieved to obtain the Japanese apricot KNOX proteins, and the HMMER software (http://hmmer.org/) was used to analyze the candidates KNOX (*E*value > 10^−5^) were screened and KNOX protein sequences were extracted using TBtools software. After removing redundant and repetitive sequences, the obtained sequences were phylogenetically analyzed using the Arabidopsis (*A. thaliana*) KNOX protein as a reference and MAFFT v7.475 software (Magnani and Hake 2008, Piazza et al. 2010, Zhang et al. 2022). The phylogenetic tree was constructed using the maximum likelihood (ML) method (bootstrap value: 1,000) to obtain phylogenetic relationships.

### RNA extraction and RT-qPCR analysis

Total RNA was extracted from frozen samples using an RNA extraction kit (Tiangen, China) and treated with RNase-free DNase I (TaKaRa, Tokyo, Japan) to reduce potential redundant genomic DNA. First-strand cDNA was generated using a reverse transcription kit (TaKaRa, Tokyo, Japan) according to the manufacturer's instructions. qPCR was conducted using a SYBR-Green PCR kit (TakaRa, Tokyo, Japan) per the manufacturer's instructions. Each sample was analyzed in 4 replicates, and the 2^−ΔΔCt^ method (Livak and Schmittgen 2001) was used to compute the relative expression levels of each gene. The primers were designed using primer-BLAST (Supplementary Table S6). All qPCR reactions were performed on a biological and technical triplicate basis, and each experiment included a non-template control.

### *PmKNAT2/6-a*, *PmKNAT2/6-b*, *PmAGL24*, and *PmLHP1* cloning and sequence analysis

The *P. mume* genome (GCF_000346735.1_P.mume_V1.0) and its annotation files were downloaded from NCBI (https://www.ncbi.nlm.nih.gov/). The hidden Markov model of KNOX (pfam03790, pfam03791, pfam03789, and pfam05920) was obtained from PFAM (http://pfam.xfam.org/) as query domains for extracting KNOXs in the *P*. *mume* local protein database. The candidate KNOXs (*E*value > 10^–5^) in *P*. *mume* were screened using HMMER software (http://hmmer.org/), and then the KNOX protein sequences were extracted using TBtools software (https://www.tbtools.com/). After removing redundant and repeated sequences, the exact sequences of KNAT2/6-a and KNAT2/6-b were obtained by phylogenetic analysis of the obtained sequences using MEGA-X software with Arabidopsis KNOXs (Gao et al. 2015) as the query sequences. The *PmAGL24* and *PmLHP1* gene reference sequences were retrieved from the National Center for Biotechnology Information database. To acquire PCR products, cloning procedures were carried out using primers (Supplementary Table S7) built using reference sequence information. The PCR products were then subcloned into the clone007 blunt simple vector and sequenced at TsingKe (Nanjing, China). The sequences collected were compared using the BioXM 2.7 program.

### Expression vector construction, plant transformation, and identification of transgenic lines

*PmAGL24* and *PmLHP1* coding sections were subcloned and placed into the p2301-35SN vector. Two target sequences spaced 786 bp apart were designed to construct the M2CRISPR vector to generate the *agl24* (*atagl24*) mutants of Arabidopsis, utilizing the CRISPR/Cas9 system (Supplementary Table S8). The flower dip technique was used to genetically modify Arabidopsis using *Agrobacterium tumefaciens* strain (EHA105 cells)-mediated means. Genomic DNA level detection, GUS staining confirmation, and RNA level detection were used to identify transgenic lines with overexpression or mutation.

### Y1H assay

The promoter sequence upstream of the *PmKNAT2/6-a* transcription start was downloaded from NCBI. The *PmKNAT2/6-a* promoter sequence was cloned using the genomic DNA of Japanese apricot as a template, and a bait plasmid (pAbAi-PmKNAT2/6-a-pro) was constructed. The bait plasmid (pAbAi-PmKNAT2/6-a-pro) and Japanese apricot yeast library plasmid were co-transformed into *Saccharomyces cerevisiae* GAL4-AbA strain (Y1HGold cells) and uniformly coated with SD/-Ura-deficient medium containing the optimum concentration of AbA for yeast growth inhibition. The screened PmAGL24 protein was cloned, and a prey plasmid (pGADT7-PmAGL24) was constructed. The PmKNAT2/6-a-pro promoter was divided into F1 and F2 bait plasmids (pAbAi-PmKNAT2/6-a-F1 and pAbAi-PmKNAT2/6-a-F2) according to the possible binding sites and transferred into yeast Y1HGold cells, respectively. The pGADT7-Rec and pGADT7-Rec containing the candidate proteins were transferred into the decoy cells and evenly coated in SD/-Leu-deficient liquid medium containing different AbA concentrations. The growth of yeast colonies confirmed the positive interactions.

### ChIP-qPCR analysis

After collecting fresh flower buds, immunoprecipitation experiments were carried out according to the instructions of the ChIP Kit-Plants-ab117137 (Abcam, Cambridge, United Kingdom). After incubation with AGL24 and H3K27me3 antibodies, DNA solutions for subsequent quantification experiments were extracted (the same method as described above). After the exact sequence of the *KNAT2/6-a* promoter was obtained by cloning and sequencing, specific primers were designed in 200 to 300 bp (Supplementary Fig. S21; Supplementary Table S9).

### FISH

FISH experiments were performed according to the previous study and images were acquired using a Zeiss LSM 800 confocal microscope (Zeiss, Germany) (Shi et al. 2023). The excitation wavelength used for the fluorescence microscope to observe the 4′,6-diamidino-2-phenylindole (DAPI) channel was 405 nm, the exposure duration was 100 ms, and the gain was 1.5×. The excitation wavelength for the red fluorescent protein (RFP) channel is 561 nm, the exposure duration is 900 ms, and the gain is 5.1×.

### Protein expression and purification

The target plasmids were constructed on pGEX-4T-1 or pCold II vectors, and the target proteins were expressed using Chemically Competent Cell BL21 (DE3) (Yeasen, Shanghai, China). After purification by adsorption of agarose gel beads (Sangong, Shanghai, China), the protein solution was eluted and dialyzed into 1 ∗ PBS solution.

### SPR and BiFC assay

The SPR experiments were performed in a BIAcore X-100 system. The PmLHP1 protein was immobilized on a CM5 sensor chip (GE Healthcare) by amine coupling. The target protein was diluted using a gradient, and kinetic data were analyzed using a steady-state affinity model using Biacore X100 evaluation software.

For BiFC analysis, the target gene plasmids were constructed into YCE and YNE vectors and co-transformed into *N. benthamiana* epidermal cells, cultured in the dark for 2 d and observed using a confocal laser scanning microscope (LSM800, Zeiss, Germany). In fluorescence microscopy observation experiments, the excitation wavelengths of the DAPI and green fluorescent protein (GFP) channels were 405 and 488 nm, respectively.

### Dual-luciferase (LUC) assay

For LUC assay, all fused reporter (p0800-LUC-*PmKNAT2/6-a*-pro) and effector (p2301-PmAGL24) plasmids were co-transformed into *A*. *tumefaciens* strain GV3101 containing pSoup-p19-#AC1003 (Weidi, Shanghai, China). *A. tumefaciens* GV3101 pSoup-p19 carrying an empty effector was used as a control. The experiment was carried out according to the instructions of the Dual Luciferase Reporter Assay Kit-#DL101-01 (Vazyme, Shanghai, China), and the values of LUC activities were recorded by the SpectraMax iD5 (TECAN, Switzerland) instrument.

### GUS immunohistochemistry assay

For GUS immunohistochemical assay, vector 35S::pBI121 and p1302-PmAGL24 were mixed and co-injected into a *N. benthamiana* leaf, and PmKNAT2/6-a-pro::pBI121 and p1302-PmAGL24 were mixed and co-injected into the other half of the same *N. benthamiana* leaf. After GUS staining (Yeasen, Shanghai, China), decolorization was performed using alcohol and documented by a photometer.

### Statistical analyses

The Student's *t*-test was performed by IBM SPSS Statistics 23 programmer (ANOVA). To discover whether the test was significantly different, a 0.05 *P*-value cutoff was used. Using GraphPad Prism 8 software, standard deviation (Sd) analysis of biological replicates of independent samples was performed, and error bars and graphs were plotted.

### Accession numbers

Sequences of PmKNAT2/6-a (LOC 103343109), PmKNAT2/6-b (LOC 103321797), PmAGL24 (LOC 103319497), and PmLHP1 (LOC 103331365) are available at NCBI.

## Supplementary Material

kiae069_Supplementary_Data

## Supplementary data

The following materials are available in the online version of this article.

**Supplementary Figure S1**. DNA sequence analysis of the *PmKNAT2/6-a* gene in ‘LY’ and ‘DY’.

**Supplementary Figure S2**. DNA sequence analysis of the *PmKNAT2/6-b* gene in ‘LY’ and ‘DY’.

**Supplementary Figure S3**. Molecular identification of *PmKNAT2/6-a* and *PmKNAT2/6-b* overexpression Arabidopsis.

**Supplementary Figure S4**. Construction of expression vector of *PmKNAT2/6-a* and *PmKNAT2/6*.

**Supplementary Figure S5**. Construction of pAbAi-*PmKNAT2/6-a*-pro vector.

**Supplementary Figure S6**. Selection of optimal concentration of AbA for yeast growth inhibition.

**Supplementary Figure S7**. PCR detection of proteins that may bind to the *PmKNAT2/6-a* promoter.

**Supplementary Figure S8**. Construction of pAbAi-*PmKNAT2/6-a*-*pro*-F1, pAbAi-*PmKNAT2/6-a*-*pro-*F2, pGADT7-*PmAGL24*, and pGADT7-*PmSOC1* vectors.

**Supplementary Figure S9**. Physical interaction of PmAGL24 with the *PmKNAT2/6-a* promoter in Y1H assay.

**Supplementary Figure S10**. Promoter sequence analysis of the *PmKNAT2/6-a* gene in ‘LY’ and ‘DY’.

**Supplementary Figure S11**. Construction of pGreen II 0800-LUC*-PmKNAT2/6-a*-pro and p2301-*PmAGL24* vectors.

**Supplementary Figure S12**. Construction of PmKNAT2/6-a-pro::pBI121 and p1302-PmAGL24 vectors.

**Supplementary Figure S13**. Molecular identification of *PmAGL24* overexpression and knockdown of *AtAGL24* in Arabidopsis.

**Supplementary Figure S14**. Phenotypic analysis of *PmAGL24* overexpression and knockdown of *AtAGL24* in Arabidopsis.

**Supplementary Figure S15**. Construction of expression vector of *PmLHP1*.

**Supplementary Figure S16**. Molecular identification of *PmLHP1* overexpression in Arabidopsis.

**Supplementary Figure S17**. Phenotypic analysis of the transgenic *PmLHP1* in Arabidopsis.

**Supplementary Figure S18**. Construction of pCold II-PmLHP1 and pGex-4T-1-PmAGL24 vectors.

**Supplementary Figure S19**. Construction of YCE-PmAGL24 and YNE-PmLHP1 vectors.

**Supplementary Figure S20**. Expression analysis of *AtKNAT2* and *AtkKNAT6* in *lhp1* mutant Arabidopsis.

**Supplementary Figure S21**. Schematic diagram of the ChIP-qPCR primer design region (H3K27me3).

**Supplementary Table S1**. Introduction of candidate genes obtained from Y1H screen libraries.

**Supplementary Table S2**. Primers design and sequences of Y1H and ChIP-qPCR (PmAGL24).

**Supplementary Table S3**. Predicted functional effects of AGL24 protein point mutations in 2 Japanese apricot.

**Supplementary Table S4**. Gray values of western blot.

**Supplementary Table S5**. Determination of cytokinin content in flower buds of Japanese apricot at the stage of pistil number differentiation.

**Supplementary Table S6**. Primer sequences used for RT-qPCR in Japanese apricot.

**Supplementary Table S7**. Primer sequences used for cloning the CDS regions in Japanese apricot.

**Supplementary Table S8**. Primer sequences used for construction of *atagl24* mutants using the CRISPR/Cas9 system in Arabidopsis.

**Supplementary Table S9**. Primer sequences used for ChIP-qPCR in Japanese apricot (H3K27me3).

## Dive Curated Terms

The following phenotypic, genotypic, and functional terms are of significance to the work described in this paper:

AGL24 Gramene: AT4G24540
AGL24 Araport: AT4G24540

KNATM Gramene: AT1G14760
KNATM Araport: AT1G14760

KNAT2 Gramene: AT1G70510
KNAT2 Araport: AT1G70510

STM Gramene: At1g62360
STM Araport: At1g62360

KNAT6 Gramene: AT1G23380
KNAT6 Araport: AT1G23380

FLC Gramene: AT5G10140
FLC Araport: AT5G10140

SVP Gramene: AT2G22540
SVP Araport: AT2G22540

LHP1 Gramene: AT5G17690
LHP1 Araport: AT5G17690

## References

[kiae069-B1] Belles-Boix E , Hamant O, Witiak SM, Morin H, Traas J, Pautot V. *KNAT6*: an Arabidopsis homeobox gene involved in meristem activity and organ separation. Plant Cell. 2006:18(8):1900–1907. 10.1105/tpc.106.041988 16798887 PMC1533965

[kiae069-B2] Bowman JL , Smyth DR, Meyerowitz EM. Genetic interactions among floral homeotic genes of Arabidopsis. Development. 1991:112(1):1–20. 10.1242/dev.112.1.1 1685111

[kiae069-B3] Byrne ME , Barley R, Curtis M, Arroyo JM, Dunham M, Hudson A, Martienssen RA. *Asymmetric leaves1* mediates leaf patterning and stem cell function in Arabidopsis. Nature. 2000:408(6815):967–971. 10.1038/35050091 11140682

[kiae069-B4] Byrne ME , Groover AT, Fontana JR, Martienssen RA. Phyllotactic pattern and stem cell fate are determined by the Arabidopsis homeobox gene *BELLRINGER*. Development. 2003:130(17):3941–3950. 10.1242/dev.00620 12874117

[kiae069-B5] Dockx J , Quaedvlieg N, Keultjes G, Kock P, Weisbeek P, Smeekens S. The homeobox gene ATK1 of *Arabidopsis thaliana* is expressed in the shoot apex of the seedling and in flowers and inflorescence stems of mature plants. Plant Mol Biol. 1995:28(4):723–737. 10.1007/BF00021196 7647303

[kiae069-B6] Douglas SJ , Chuck G, Dengler RE, Pelecanda L, Riggs CD. KNAT1 and ERECTA regulate inflorescence architecture in Arabidopsis. Plant Cell. 2002:14(3):547–558. 10.1105/tpc.010391 11910003 PMC150578

[kiae069-B7] Gallois JL , Woodward C, Reddy GV, Sablowski R. Combined SHOOT MERISTEMLESS and WUSCHEL trigger ectopic organogenesis in Arabidopsis. Development. 2002:129(13):3207–3217. 10.1242/dev.129.13.3207 12070095

[kiae069-B8] Gao J , Yang X, Zhao W, Lang T, Samuelsson T. Evolution, diversification, and expression of KNOX proteins in plants. Front Plant Sci. 2015:23(6):882. 10.3389/fpls.2015.00882 PMC4617109 26557129

[kiae069-B9] Gregis V , Sessa A, Colombo L, Kater MM. *AGL24*, *SHORT VEGETATIVE PHASE*, and *APETALA1* redundantly control *AGAMOUS* during early stages of flower development in Arabidopsis. Plant Cell. 2006:18(6):1373–1382. 10.1105/tpc.106.041798 16679456 PMC1475495

[kiae069-B10] Gregis V , Sessa A, Dorca-Fornell C, Kater MM. The Arabidopsis floral meristem identity genes AP1, AGL24 and SVP directly repress class B and C floral homeotic genes. Plant J. 2009:60(4):626–637. 10.1111/j.1365-313X.2009.03985.x 19656343

[kiae069-B11] Guo M , Thomas J, Collins G, Timmermans MC. Direct repression of KNOX loci by the ASYMMETRIC LEAVES1 complex of Arabidopsis. Plant Cell. 2008:20(1):48–58. 10.1105/tpc.107.056127 18203921 PMC2254922

[kiae069-B12] Hamant O , Nogué F, Belles-Boix E, Jublot D, Grandjean O, Traas J, Pautot V. The KNAT2 homeodomain protein interacts with ethylene and cytokinin signaling. Plant Physiol. 2002:130(2):657–665. 10.1104/pp.004564 12376633 PMC166595

[kiae069-B13] Hay A , Tsiantis M. KNOX genes: versatile regulators of plant development and diversity. Development. 2010:137(19):3153–3165. 10.1242/dev.030049 20823061

[kiae069-B14] Jasinski S , Piazza P, Craft J, Hay A, Woolley L, Rieu I, Phillips A, Hedden P, Tsiantis M. KNOX action in Arabidopsis is mediated by coordinate regulation of cytokinin and gibberellin activities. Curr Biol. 2005:15(17):1560–1565. 10.1016/j.cub.2005.07.023 16139211

[kiae069-B15] Keren-Keiserman A , Shtern A, Levy M, Chalupowicz D, Furumizu C, Alvarez JP, Amsalem Z, Arazi T, Alkalai-Tuvia S, Efroni I, et al *CLASS-II* KNOX genes coordinate spatial and temporal ripening in tomato. Plant Physiol. 2022:190(1):657–668. 10.1093/plphys/kiac290 35703985 PMC9434150

[kiae069-B16] Kerstetter R , Vollbrecht E, Lowe B, Veit B, Yamaguchi J, Hake S. Sequence analysis and expression patterns divide the maize knotted1-like homeobox genes into two classes. Plant Cell. 1994:6(12):1877–1887. 10.1105/tpc.6.12.1877 7866030 PMC160568

[kiae069-B17] Kitamura Y , Takeuchi T, Yamane H, Tao R. Simultaneous down-regulation of *Dormancy-associated Mads-box6* and *SOC1* during dormancy release in Japanese apricot (*Prunus mume*) flower buds. J Hortic Sci Biotech. 2016:91(5):476–482. 10.1080/14620316.2016.1173524

[kiae069-B18] Lee J , Oh M, Park H, Lee I. SOC1 translocated to the nucleus by interaction with AGL24 directly regulates leafy. Plant J. 2008:55(5):832–843. 10.1111/j.1365-313X.2008.03552.x 18466303

[kiae069-B19] Lenhard M , Bohnert A, Jürgens G, Laux T. Termination of stem cell maintenance in Arabidopsis floral meristems by interactions between *WUSCHEL* and *AGAMOUS*. Cell. 2001:105(6):805–814. 10.1016/s0092-8674(01)00390-7 11440722

[kiae069-B20] Li E , Bhargava A, Qiang W, Friedmann MC, Forneris N, Savidge RA, Johnson LA, Mansfield SD, Ellis BE, Douglas CJ. The Class II KNOX gene KNAT7 negatively regulates secondary wall formation in Arabidopsis and is functionally conserved in Populus. New Phytol. 2012a:194(1):102–115. 10.1111/j.1469-8137.2011.04016.x 22236040

[kiae069-B21] Li Z , Li B, Shen WH, Huang H, Dong A. TCP transcription factors interact with AS2 in the repression of class-I KNOX genes in *Arabidopsis thaliana*. Plant J. 2012b:71(1):99–107. 10.1111/j.1365-313X.2012.04973.x 22380849

[kiae069-B22] Lincoln C , Long J, Yamaguchi J, Serikawa K, Hake S. A knotted1-like homeobox gene in Arabidopsis is expressed in the vegetative meristem and dramatically alters leaf morphology when overexpressed in transgenic plants. Plant Cell. 1994:6(12):1859–1876. 10.1105/tpc.6.12.1859 7866029 PMC160567

[kiae069-B23] Liu C , Xi W, Shen L, Tan C, Yu H. Regulation of floral patterning by flowering time genes. Dev Cell. 2009:16(5):711–722. 10.1016/j.devcel.2009.03.011 19460347

[kiae069-B24] Liu X , Gao L, Dinh TT, Shi T, Li D, Wang R, Guo L, Xiao L, Chen X. DNA topoisomerase I affects Polycomb group protein-mediated epigenetic regulation and plant development by altering nucleosome distribution in Arabidopsis. Plant Cell. 2014:26(7):2803–2817. 10.1105/tpc.114.124941 25070639 PMC4145115

[kiae069-B25] Livak KJ , Schmittgen TD. Analysis of relative gene expression data using real-time quantitative PCR and the 2^−ΔΔCT^method. Methods. 2001:25(4):402–408. 10.1006/meth.2001.1262 11846609

[kiae069-B26] Magnani E , Hake S. KNOX lost the OX: the Arabidopsis KNATM gene defines a novel class of KNOX transcriptional regulators missing the homeodomain. Plant Cell. 2008:20(4):875–887. 10.1105/tpc.108.058495 18398054 PMC2390742

[kiae069-B27] Margueron R , Reinberg D. The Polycomb complex PRC2 and its mark in life. Nature. 2011:469(7330):343–349. 10.1038/nature09784 21248841 PMC3760771

[kiae069-B28] Michaels SD , Ditta G, Gustafson-Brown C, Pelaz S, Yanofsky M, Amasino RM. AGL24 acts as a promoter of flowering in Arabidopsis and is positively regulated by vernalization. Plant J. 2003:33(5):867–874. 10.1046/j.1365-313x.2003.01671.x 12609028

[kiae069-B29] Müller-Xing R , Ardiansyah R, Xing Q, Faivre L, Tian J, Wang G, Zheng Y, Wang X, Jing T, de Leau E, et al Polycomb proteins control floral determinacy by H3K27me3-mediated repression of pluripotency genes in *Arabidopsis thaliana*. J Exp Bot. 2022:73(8):2385–2402. 10.1093/jxb/erac013 35045165

[kiae069-B30] Pautot V , Dockx J, Hamant O, Kronenberger J, Grandjean O, Jublot D, Traas J. KNAT2: evidence for a link between knotted-like genes and carpel development. Plant Cell. 2001:13(8):1719–1734. 10.1105/tpc.010184 11487688 PMC139140

[kiae069-B31] Piazza P , Bailey CD, Cartolano M, Krieger J, Cao J, Ossowski S, Schneeberger K, He F, de Meaux J, Hall N, et al *Arabidopsis thaliana* leaf form evolved via loss of KNOX expression in leaves in association with a selective sweep. Curr Biol. 2010:20(24):2223–2228. 10.1016/j.cub.2010.11.037 21129970

[kiae069-B32] Ragni L , Belles-Boix E, Günl M, Pautot V. Interaction of *KNAT6* and *KNAT2* with *BREVIPEDICELLUS* and *PENNYWISE* in Arabidopsis inflorescences. Plant Cell. 2008:20(4):888–900. 10.1105/tpc.108.058230 18390591 PMC2390738

[kiae069-B33] Schneitz K . The molecular and genetic control of ovule development. Curr Opin Plant Biol. 1999:2(1):13–17. 10.1016/s1369-5266(99)80003-x 10047571

[kiae069-B34] Scofield S , Dewitte W, Murray JA. The *KNOX* gene *SHOOT MERISTEMLESS* is required for the development of reproductive meristematic tissues in Arabidopsis. Plant J. 2007:50(5):767–781. 10.1111/j.1365-313X.2007.03095.x 17461793

[kiae069-B35] Scofield S , Murray JA. *KNOX* gene function in plant stem cell niches. Plant Mol Biol. 2006:60(6):929–946. 10.1007/s11103-005-4478-y 16724262

[kiae069-B36] Shi T , Bai Y, Wu X, Wang Y, Iqbal S, Tan W, Ni Z, Gao Z. PmAGAMOUS recruits Polycomb protein PmLHP1 to regulate single-pistil morphogenesis in Japanese apricot. Plant Physiol. 2023:193(1):466–482. 10.1093/plphys/kiad292 37204822

[kiae069-B37] Shim D , Ko JH, Kim WC, Wang Q, Keathley DE, Han KH. A molecular framework for seasonal growth-dormancy regulation in perennial plants. Hortic Res. 2014:1:14059. 10.1038/hortres.2014.59 26504555 PMC4591672

[kiae069-B38] Tolhuis B , de Wit E, Muijrers I, Teunissen H, Talhout W, van Steensel B, van Lohuizen M. Genome-wide profiling of PRC1 and PRC2 Polycomb chromatin binding in *Drosophila melanogaster*. Nat Genet. 2006:38(6):694–699. 10.1038/ng1792 16628213

[kiae069-B39] Turck F , Roudier F, Farrona S, Martin-Magniette ML, Guillaume E, Buisine N, Gagnot S, Martienssen RA, Coupland G, Colot V. Arabidopsis TFL2/LHP1 specifically associates with genes marked by trimethylation of histone H3 lysine 27. PLoS Genet. 2007:3(6):e86. 10.1371/journal.pgen.0030086 17542647 PMC1885283

[kiae069-B40] Wu X , Shi T, Iqbal S, Zhang Y, Liu L, Gao Z. Genome-wide discovery and characterization of flower development related long non-coding RNAs in *Prunus mume*. BMC Plant Biol. 2019:19(1):64. 10.1186/s12870-019-1672-7 30744565 PMC6371585

[kiae069-B41] Yamane H , Wada M, Honda C, Matsuura T, Ikeda Y, Hirayama T, Osako Y, Gao-Takai M, Kojima M, Sakakibara H, et al Overexpression of *Prunus DAM6* inhibits growth, represses bud break competency of dormant buds and delays bud outgrowth in apple plants. PLoS One. 2019:14(4):e0214788. 10.1371/journal.pone.0214788 30964897 PMC6456227

[kiae069-B42] Yu H , Ito T, Wellmer F, Meyerowitz EM. Repression of AGAMOUS-LIKE 24 is a crucial step in promoting flower development. Nat Genet. 2004:36(2):157–161. 10.1038/ng1286 14716314

[kiae069-B43] Zhang X , Clarenz O, Cokus S, Bernatavichute YV, Pellegrini M, Goodrich J, Jacobsen SE. Whole-genome analysis of histone H3 lysine 27 trimethylation in Arabidopsis. PLoS Biol. 2007:5(5):e129. 10.1371/journal.pbio.0050129 17439305 PMC1852588

[kiae069-B44] Zhang Y , Yin Q, Qin W, Gao H, Du J, Chen J, Li H, Zhou G, Wu H, Wu AM. The Class II KNOX family members *KNAT3* and *KNAT7* redundantly participate in Arabidopsis seed coat mucilage biosynthesis. J Exp Bot. 2022:73(11):3477–3495. 10.1093/jxb/erac066 35188965

