# Supplementary material for: AGAMOUS-LIKE24 controls pistil number in Japanese apricot by targeting the KNOTTED1-LIKE gene KNAT2/6-a
Source: Plant Physiol. 2024 Feb 12;195(1):566–79. doi: 10.1093/plphys/kiae069 (PMC11060673; doi:10.1093/plphys/kiae069)
Supplement: kiae069_Supplementary_Data [file kiae069_supplementary_data.pdf]

Supplemental Figure S1. DNA sequence analysis of the *PmKNAT2/6-a* gene in LY and DY.

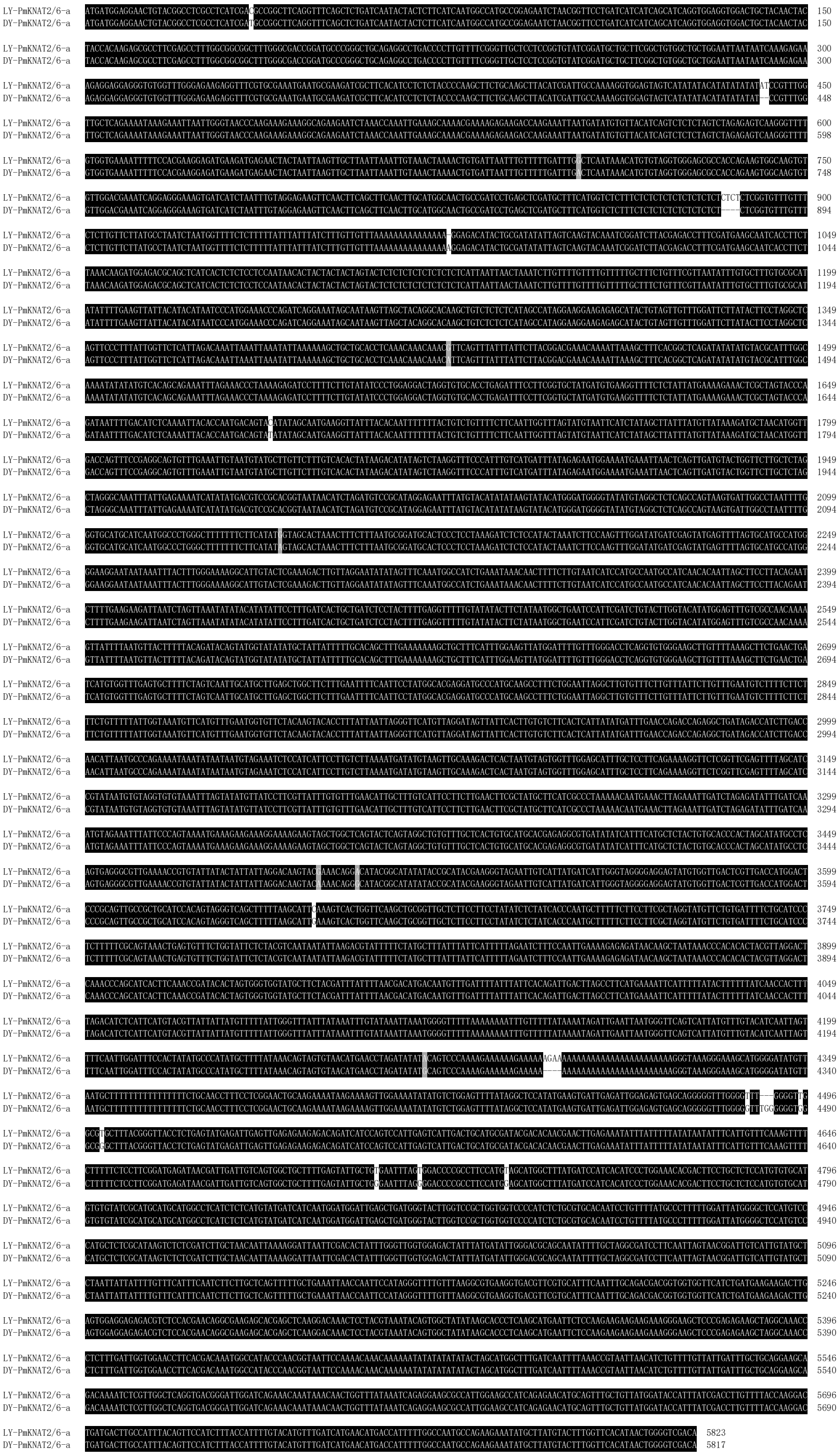

Black background represents base identity.

Supplemental Figure S2. DNA sequence analysis of the *PmKNAT2/6-b* gene in LY and DY.

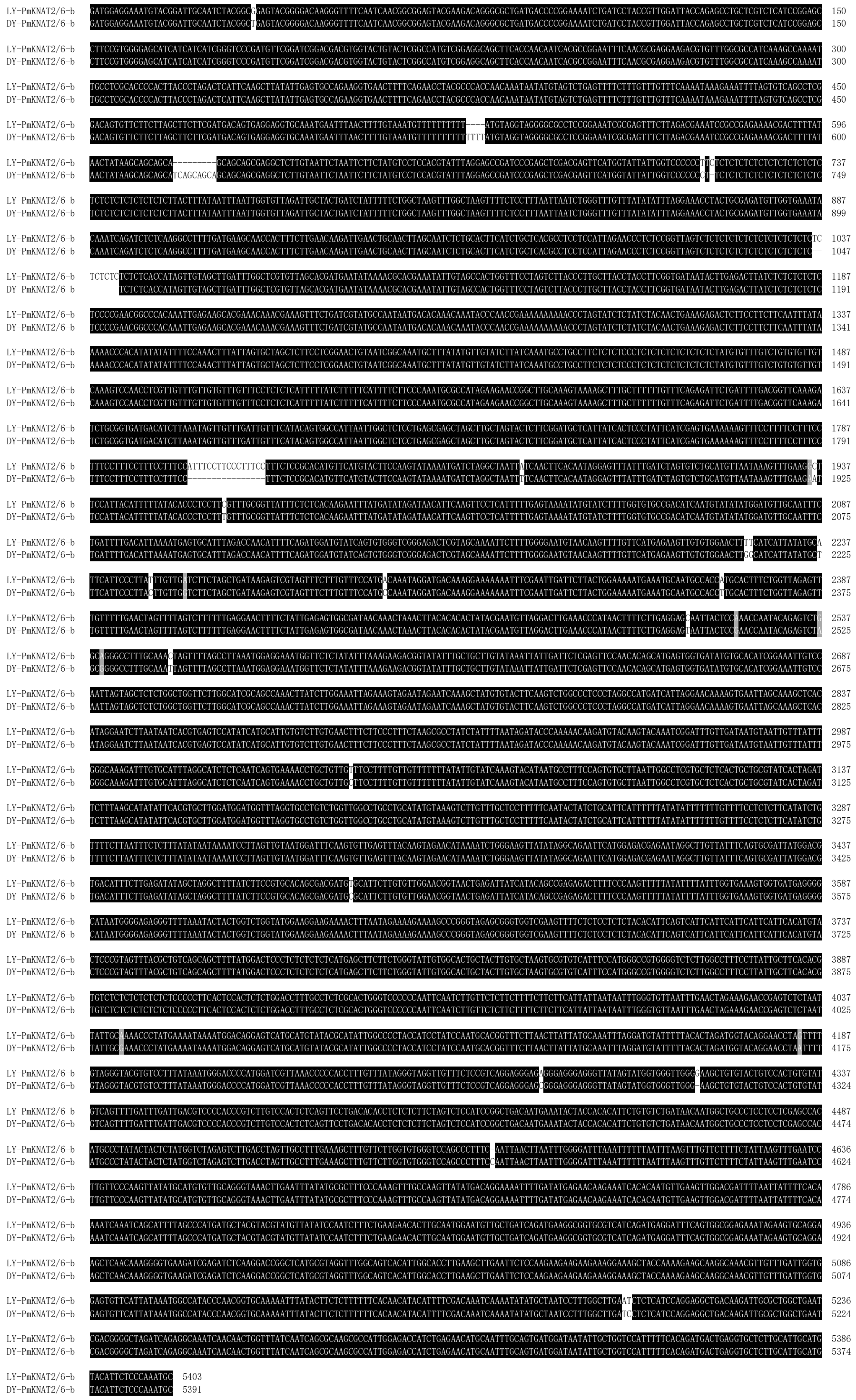

Black background represents base identity.

Supplemental Figure S3. Molecular identification of *PmKNAT2/6-a and PmKNAT2/6-b* overexpression Arabidopsis.

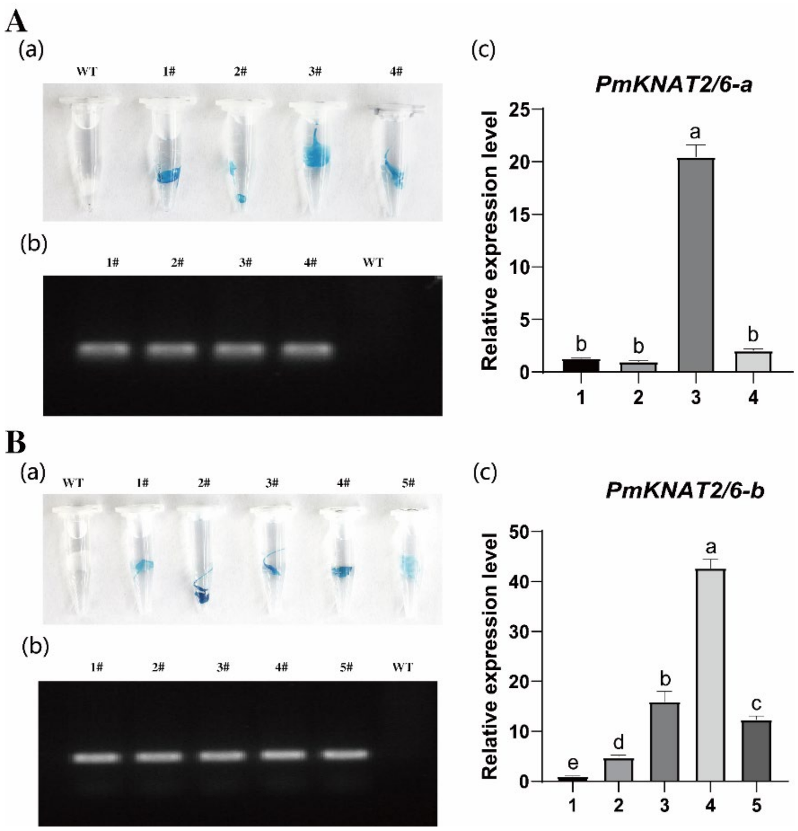

(A) The identification of *PmKNAT2/6-a* overexpressing Arabidopsis lines. GUS staining (a), detection of target gene insertion into the genome(b) and expression of *PmAGL24* in transgenic lines (c). Letters indicate significant changes in ANOVA ($P < 0.05$). **(B)** The identification of *PmKNAT2/6-b* overexpressing Arabidopsis lines. GUS staining (a), detection of target gene insertion into the genome(b) and expression of *PmAGL24* in transgenic lines (c). Error bars represent SE based on 3 biological replicates. Letters indicate significant differences using Student's *t*-test ($p < 0.05$). The figures on the Y-axis represent the transgenic plant numbers of the different lines.

Supplemental Figure S4. Construction of expression vector of *PmKNAT2/6-a* and *PmKNAT2/6*.

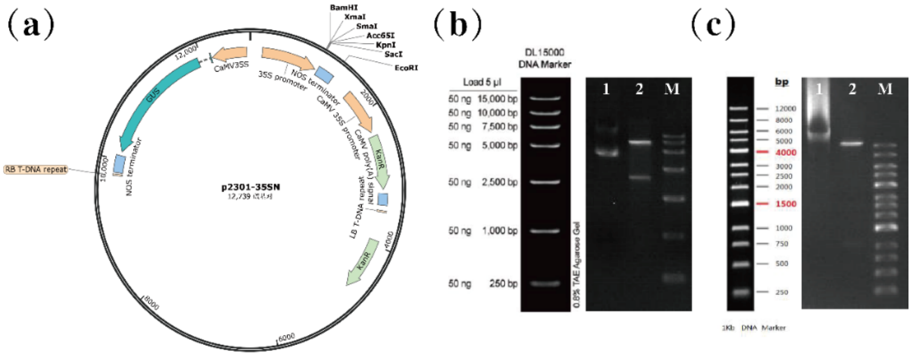

(a) Vector mapping of p2301-35SN. (b) Construction of p2301-*PmKNAT2/6-a* vector. Primers: F- CAGATCTGATGATGGAGGAACTGTACGGC;R- TCAGTCCTTGGTAAAACAAGGCAGATCTG. M is Marker; lane 1 is p2301-*PmKNAT2/6-a* vector plasmid; lane 2 is enzyme digestion assay (restriction enzyme: *BglII*). (c) Construction of p2301-*PmKNAT2/6-b* vector. Primers: F- CGGATCCGATGGAGGAAATGTACGGATTGC; R- TCAGTCATCTGTGAAAAATGGGGGTACCC. M is Marker; lane 1 is p2301-*PmKNAT2/6-b* vector plasmid; lane 2 is enzyme digestion assay (restriction enzyme: *BamHI-KpnI*).

Supplemental Figure S5. Construction of pAbAi-*PmKNAT2/6-a*-pro vector.

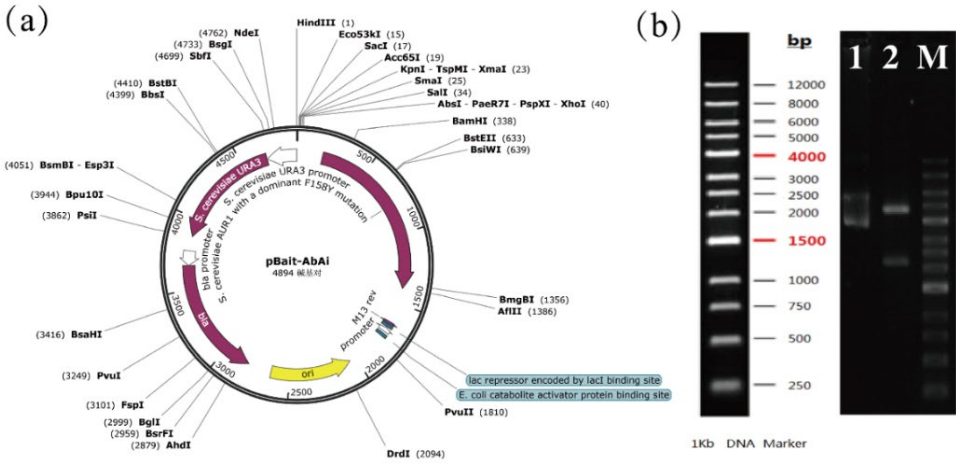

(a) Vector mapping of pBait-AbAi. (b) Construction of pAbAi-*PmKNAT2/6-a*-pro vector. Primers: F- CGAGCTCGGTATGATATGAATGCATAAATGGGCC; R- CGAGGCCGTACAGTTCCTCCATCATCCTCGAGG. M is Marker; lane 1 is pAbAi-*PmKNAT2/6-a*-pro vector plasmid; lane 2 is enzyme digestion assay (restriction enzyme: *SacI*-*XhoI*).

Supplemental Figure S6. Selection of optimal concentration of AbA for yeast growth inhibition.

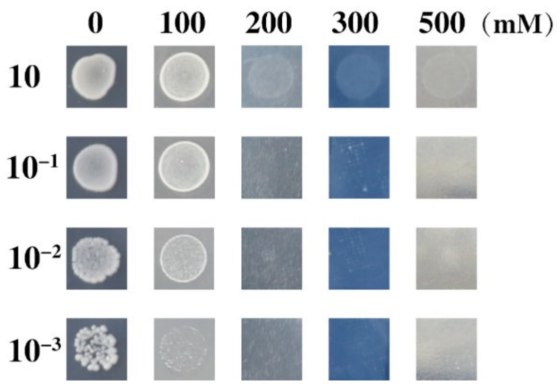

The abscissa is the concentration gradient of AbA (mM) and the coordinate is the dilution gradient of the yeast suspension. The linearized pAbAi-PmKNAT2/6-a-pro vector and pGADT7-Rec empty were cotransformed into yeast Y1HGold sensory state for AbA concentration screening. After 3 d of incubation, it was found that the 200 mM concentration of AbA could effectively inhibit the growth of yeast colonies on the defective medium of SD/-Ura at the optimal concentration.

Supplemental Figure S7. PCR detection of proteins that may bind to the *PmKNAT2/6-a* promoter.

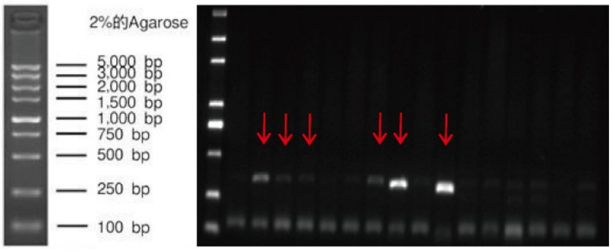

Co-transformation of the bait plasmid pAbAi-PmKNAT2/6-a-pro and library plasmid AD-cDNA in yeast receptor Y1H Gold yielded a total of 16 colonies of proteins that may bind to the PmKNAT2/6-a promoter. Six yeast fluids PCR detected as single and bright bands of monoclonal yeast were sent for sequencing. Delivery strips are indicated with red arrows.

Supplemental Figure S8. Construction of pAbAi-*PmKNAT2/6-a-pro*-F1, pAbAi-*PmKNAT2/6-a-pro*-F2, pGADT7-*PmAGL24* and pGADT7-*PmSOC1* vector.

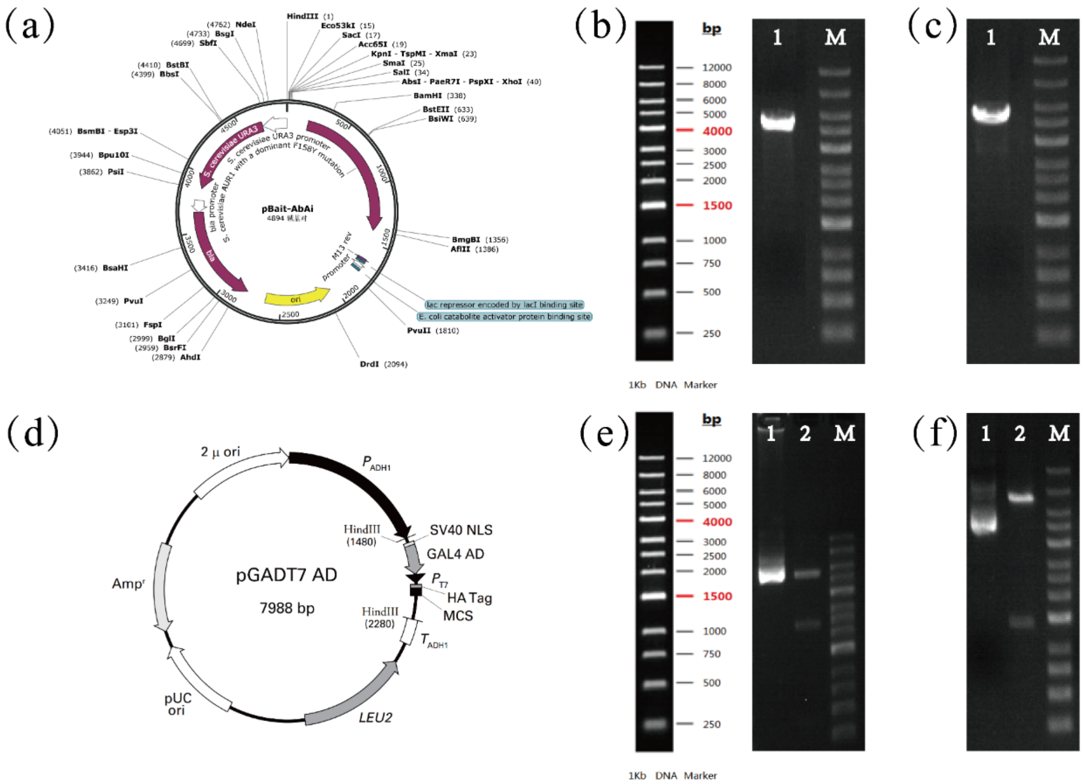

(a) Vector mapping of pBait-AbAi; (b) Construction of pAbAi-*PmKNAT2/6-a-pro*-F1 vector. Primers: F- CGAGCTCGGGCCTAAGGTTGTTTTTAATTTGGG; R- CGAGCCTTGGCTCTGTTCACCCCTCGAGG. (c) Construction of pAbAi-*PmKNAT2/6-a-pro-F2* vector. Primers: F- CTCTGGTGAACAGAGCCAAG; R- GGCCGTACAGTTCCTCCATC. (b and c) M is Marker; lane 1 are enzyme digestion assay (restriction enzyme: *SacI-XhoI*). (d) Vector mapping of pGADT7-Rec. Primers: F- CGAGCTCGATGGAGGAAATGTACGGATTGC; R- TCAGTCATCTGTGAAAAATGGCCTCGAGG. (e) Construction of pGADT7-*PmAGL24* vector. Primers: F- CCTCGAGGATGGTGAAAATGATGAGGGAGAAGA; R- CTAGGGAAGCCCCAGTTTGAGAGGGCTAGCC. (f) Construction of pGADT7-*PmSOC1* vector. Primers: F- CAAGCTTGATGGTGAGAGGAAAAACCCAGATGA; R- CTAGCGCTTTCTTCTTTCTGGCAGTCAAGCTTG. (e and f) M is Marker; lane 1 are vector plasmids; lane 2 are enzyme digestion assay (restriction enzyme: left is *XhoI-NheI*, right is *HindIII*).

Supplemental Figure S9. Physical interaction of PmAGL24 with the *PmKNAT2/6-a* promoter in Y1H assay.

In Rosaceae, MADS-box proteins are mostly associated with plant dormancy and also have some association with flower development. So, we performed point-to-point validation for PmAGL24 and PmSOC1 and found that only PmAGL24 can bind to the promoter of *PmKNAT2/6-a*.

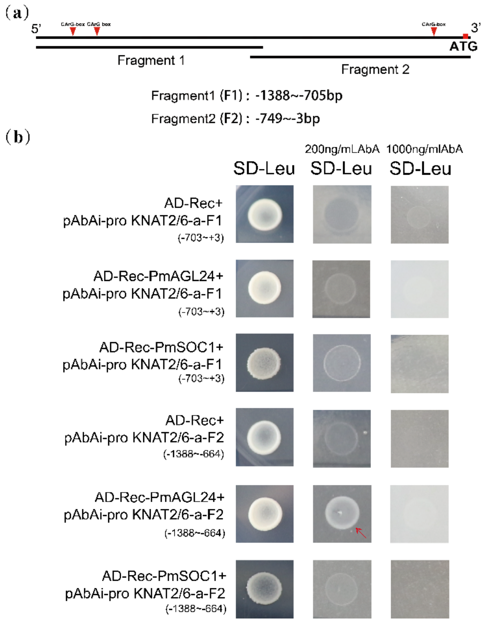

(a) The promoter of *PmKNAT2/6-a* was divided into fragment 1 (F1) and fragment 2 (F2). (b) The yeast co-transformed with different constructs were grown on SD/-Leu medium with or without AbA. The horizontal lines represent the fragment range. AD-Rec-PmAGL24 and AD-Rec-PmSOC1 were used as the prey, pAbAi-*PmKNAT2/6-a-pro*-F1 and pAbAi-*PmKNAT2/6-a-pro*-F2 as the baits, and the AD-Rec as control. The red arrow marks the normal growth of yeast colonies.

Supplemental Figure S10. Promoter sequence analysis of the *PmKNAT2/6-a* gene in LY and DY.

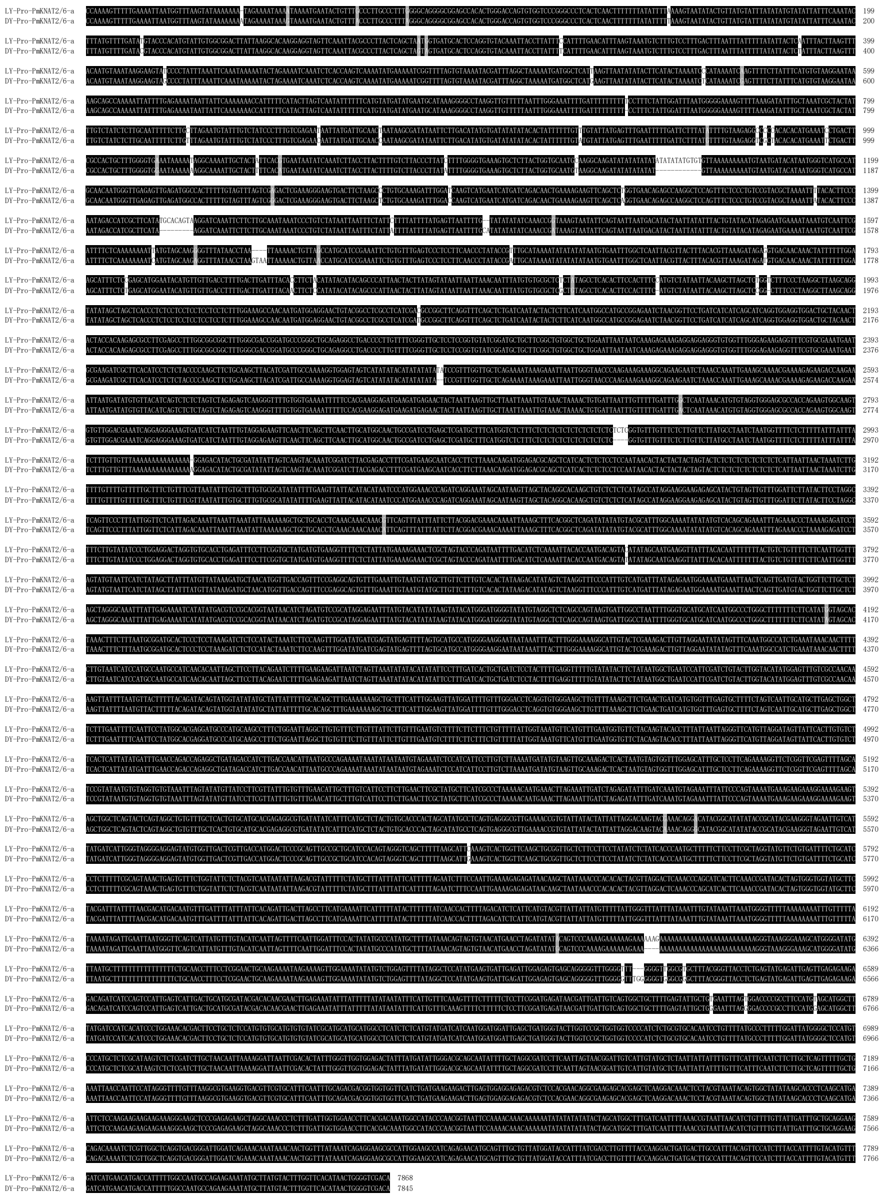

Black background represents base identity.

Supplemental Figure S11. Construction of pGreen II 0800-LUC-*PmKNAT2/6-a*-pro and p2301-*PmAGL24* vector.

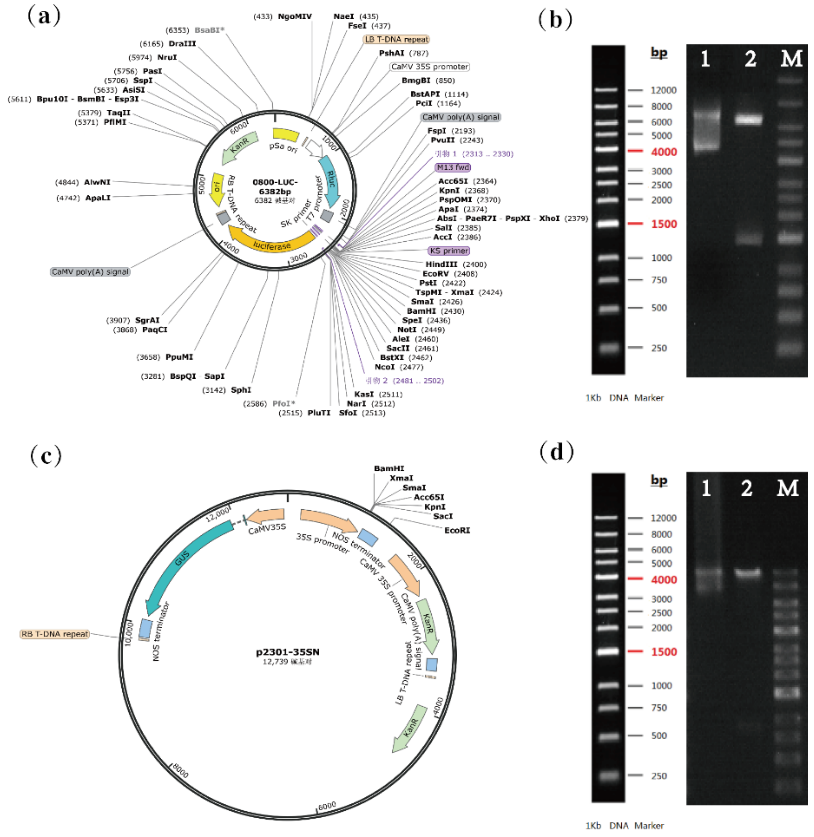

(a) Vector mapping of pGreen II 0800-LUC; (b) Construction of pGreen II 0800-LUC-*PmKNAT2/6-a*-pro. Primers: F- CCTCGAGGGTATGATATGAATGCATAAATGGGCC; R-CGAGGCCGTACAGTTCCTCCATCATTTGCGGCCGCAA. M is Marker; lane 1 is pGreen II 0800-LUC-*PmKNAT2/6-a*-pro vector plasmid; lane 2 is enzyme digestion assay (restriction enzyme: *XhoI-NotI*); (c) Vector mapping of p2301-35SN; (d) Construction of p2301-*PmAGL24* vector. Primers: F- CGGATCCGATGGTGAAAATGATGAGGGAGAAGA; R-CTAGGGAAGCCCCAGTTTGAGAGGGAATTCC. M is Marker; lane 1 is p2301-*PmAGL24* vector plasmid; lane 2 is enzyme digestion assay (restriction enzyme: *BamHI-EcoRI*).

Supplemental Figure S12. Construction of PmKNAT2/6-a-pro::pBI121 and p1302-PmAGL24 vector.

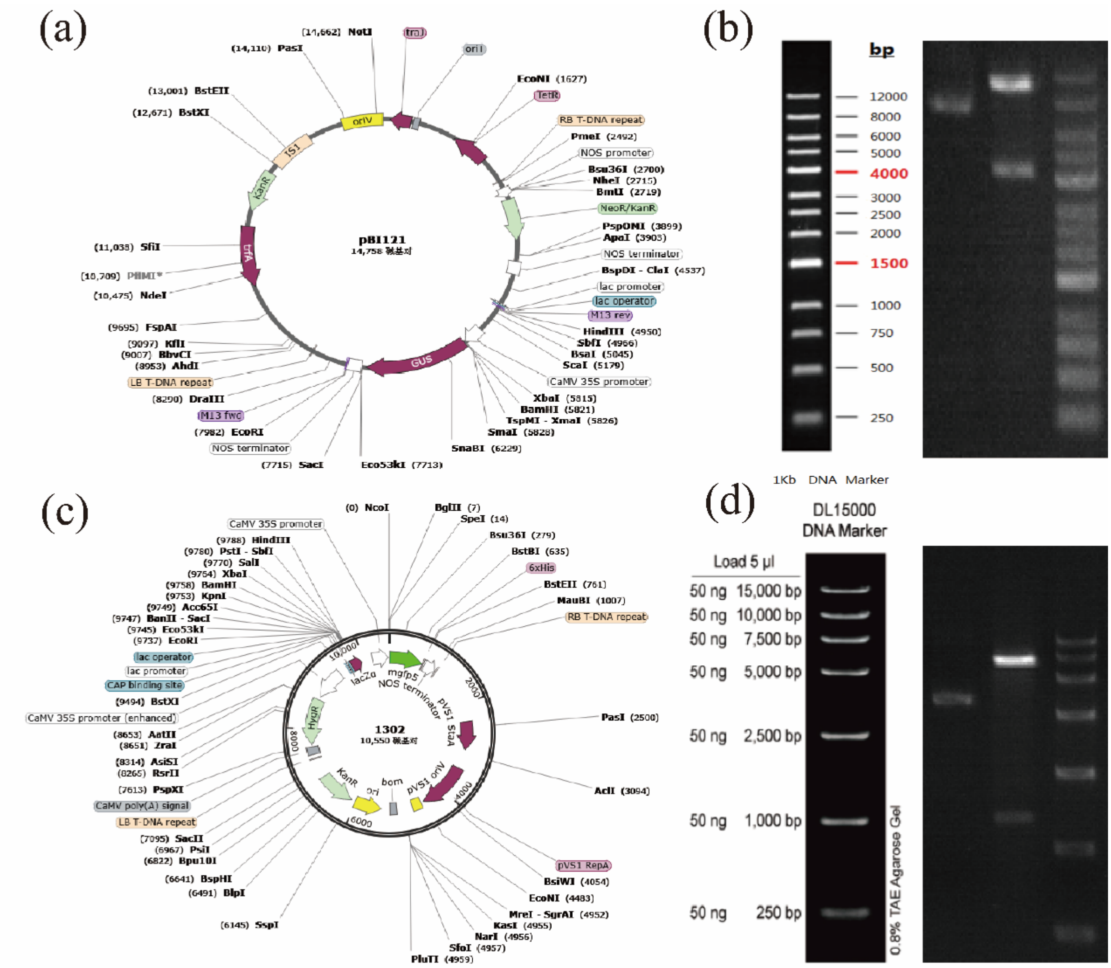

(a) Vector mapping of pBI121; (b) Construction of PmKNAT2/6-a-pro::pBI121. Primers: F-CGAGCTCGGTATGATATGAATGCATAAATGGGCC; R-CGAGGCCGTACAGTTCCTCCATCATGGGCCC. M is Marker; lane 1 is PmKNAT2/6-a-pro::pBI121 vector plasmid; lane 2 is enzyme digestion assay (restriction enzyme: *SacI-ApaI*); (c) Vector mapping of pCAMBIA1302; (d) Construction of p1302-PmAGL24 vector. Primers: F- CAAGCTTGATGGTGAAAATGATGAGGGAGAAGA; R-CTAGGGAAGCCCCAGTTTGAGAGGACTAGTC. M is Marker; lane 1 is p1302-PmAGL24 vector plasmid; lane 2 is enzyme digestion assay (restriction enzyme: *HindIII-SpeI*).

Supplemental Figure S13. Molecular identification of *PmAGL24* overexpression and knockdown of *AtAGL24* in Arabidopsis.

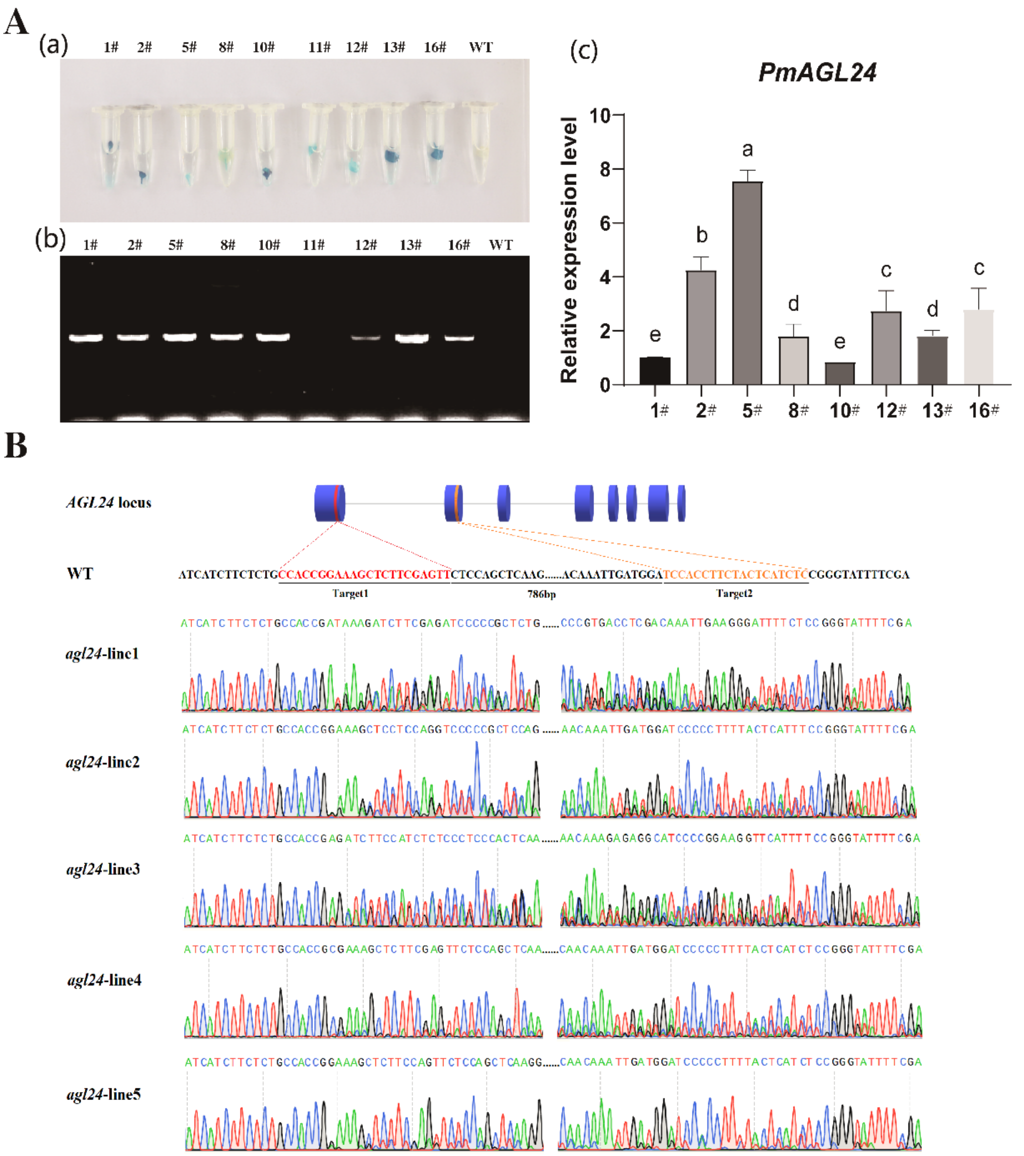

(A) The identification of *PmAGL24* overexpressing Arabidopsis lines. GUS staining (a), detection of target gene insertion into the genome(b) and expression of *PmAGL24* in transgenic lines (c). Error bars represent SE based on 3 biological replicates. Letters indicate significant differences using Student's *t*-test ($p < 0.05$). The figures on the Y-axis represent the transgenic plant numbers of the different lines. **(B)** Genotype of mutations in the *Atsoc1* locus generated by the CRISPR/Cas9 genome editing system. Two target sequences were designed to specifically target. The mutant sequences were compared with the original sequences to show the mutation. The red and orange letters indicate the sequences of targets 1 and 2, respectively.

Supplemental Figure S14. Phenotypic analysis of *PmAGL24* overexpression and knockdown of *AtAGL24* in Arabidopsis.

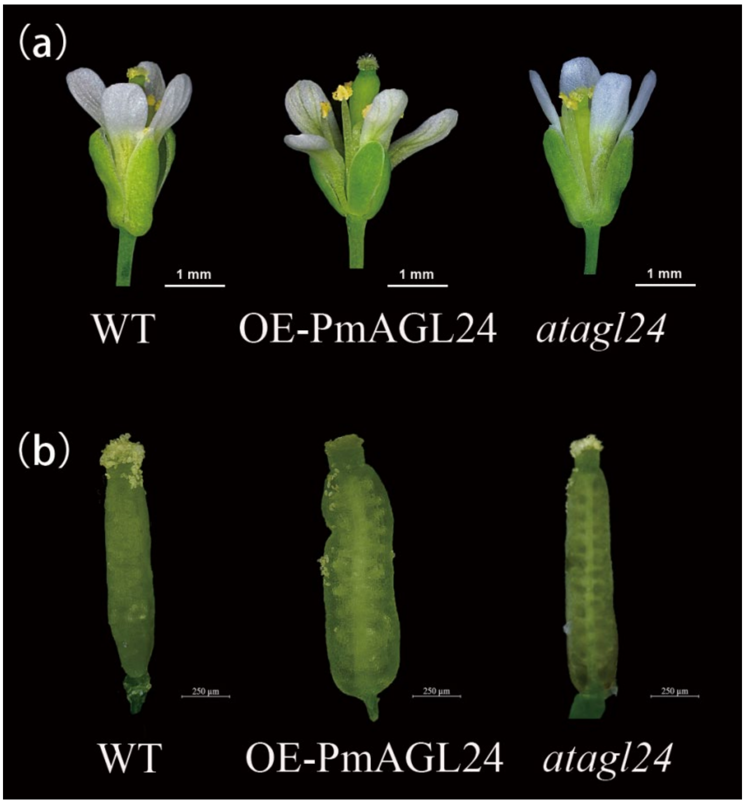

(a) is flowers of Arabidopsis and (b) is carpels of Arabidopsis. OE-PmAGL24 is the transgenic of Arabidopsis, *atagl24* is the mutant of Arabidopsis and WT is the wild type of Arabidopsis. Images were digitally extracted for comparison.

Supplemental Figure S15. Construction of expression vector of *PmLHP1*.

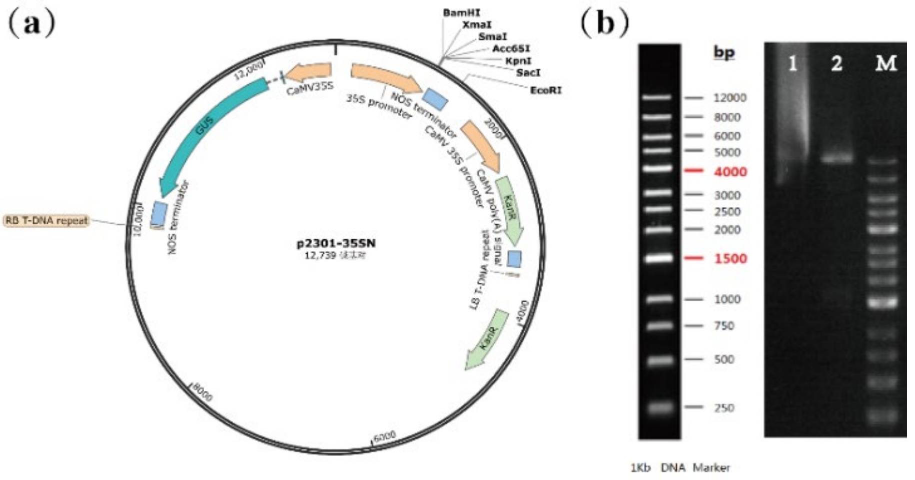

(a) Vector mapping of p2301-36SN. (b) Construction of p2301-*PmLHP1* vector. Primers: F-GGCTAGCCATGAAAGTGAAGGGAGGAGGAAG; R-TTACAATGTAGAATTGTACCGGCGGATCCG. M is Marker; lane 1 is p2301-*PmLHP1* vector plasmid; lane 2 is enzyme digestion assay (restriction enzyme: *NheI-BamHI*).

Supplemental Figure S16. Molecular identification of *PmLHP1* overexpression in Arabidopsis.

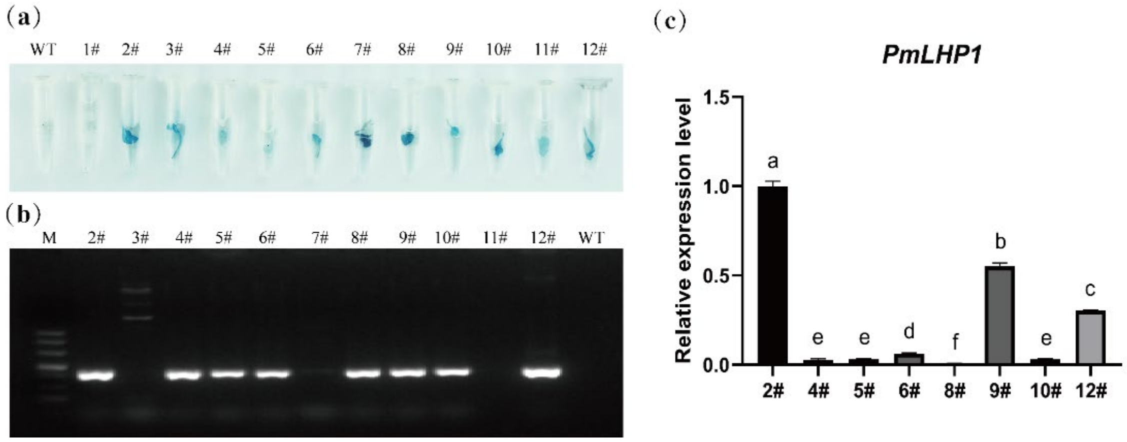

The identification of *PmLHP1*overexpressing Arabidopsis lines. (a) GUS staining, (b) detection of whether the *PmLHP1* was inserted into the genome, and (c) detection of *PmLHP1* expression in transgenic lines. Error bars represent SE based on 3 biological replicates. Letters indicate significant differences using Student's *t*-test ($p < 0.05$). The figures on the Y-axis represent the transgenic plant numbers of the different lines.

Supplemental Figure S17. Phenotypic analysis of the transgenic *PmLHP1* in Arabidopsis.

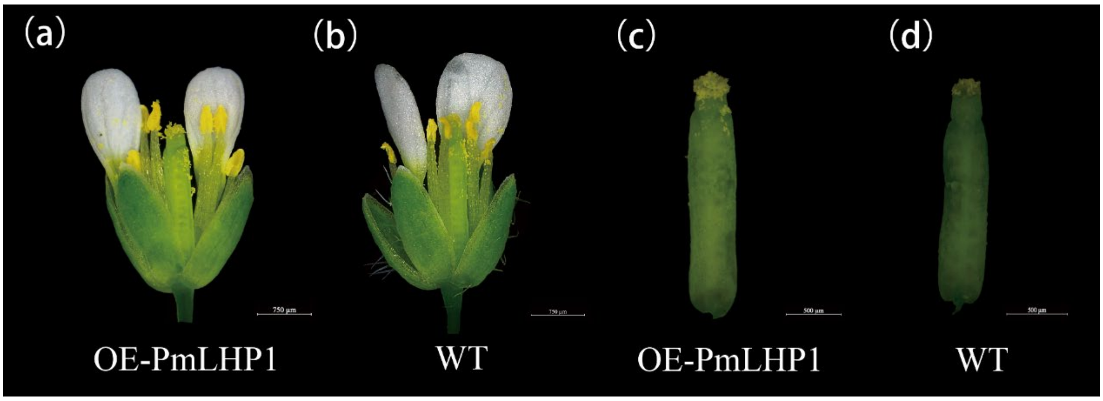

(a, b) are flowers of Arabidopsis and (c, d) are carpels of Arabidopsis. OE-PmLHP1 is the transgenic of Arabidopsis and WT is the wild type of Arabidopsis. Images were digitally extracted for comparison.

Supplemental Figure S18. Construction of pCold II-PmLHP1 and pGex-4T-1-PmAGL24 vector.

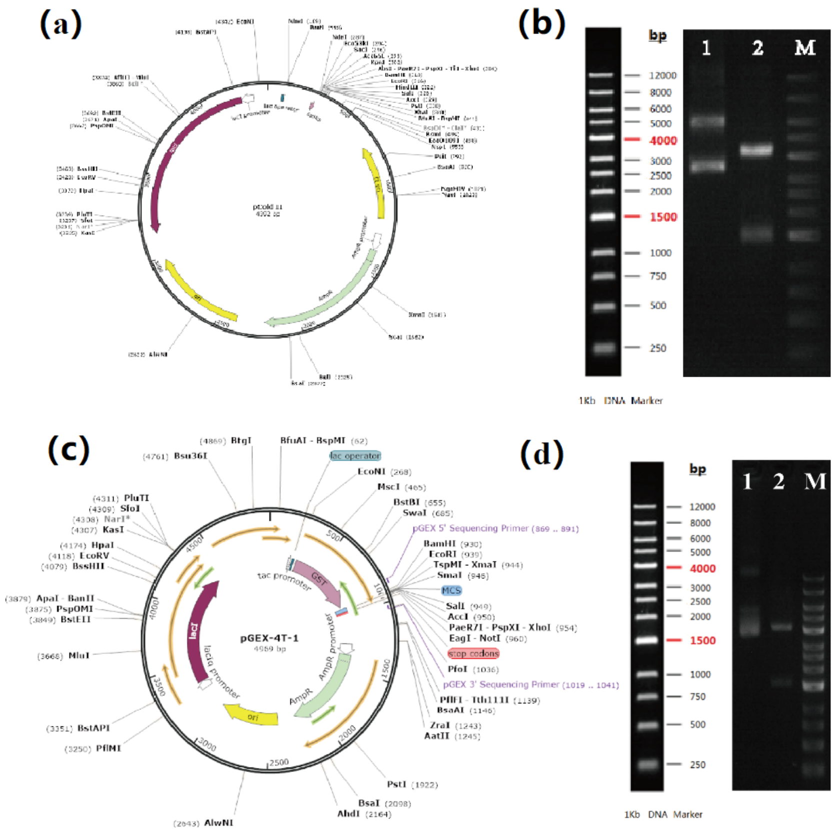

(a) Vector mapping of pCold II. (b) Construction of pCold II-*PmLHP1* vector. M is Marker; lane 1 is pCold II-*PmLHP1* vector plasmid; lane 2 is enzyme digestion assay (restriction enzyme: *NheI-BamHI*). Primers: F- GGCTAGCCATGAAAGTGAAGGGAGGAGGAAG; R- TTACAATGTAGAATTGTACCGGCGGATCCG. (c) Vector mapping of pGex-4T-1. (d) Construction of pGex-4T-1-*PmAGL24* vector. Primers: F- GAGTACTCATGGTGAAAATGATGAGGGAGAAGA; R- CTAGGGAAGCCCCAGTTTGAGAGGAGTACTC. M is Marker; lane 1 is pGex-4T-1-*PmAGL24* vector plasmid; lane 2 is enzyme digestion assay (restriction enzyme: *ScaI*).

Supplemental Figure S19. Construction of YCE-PmAGL24 and YNE-PmLHP1 vector. vector.

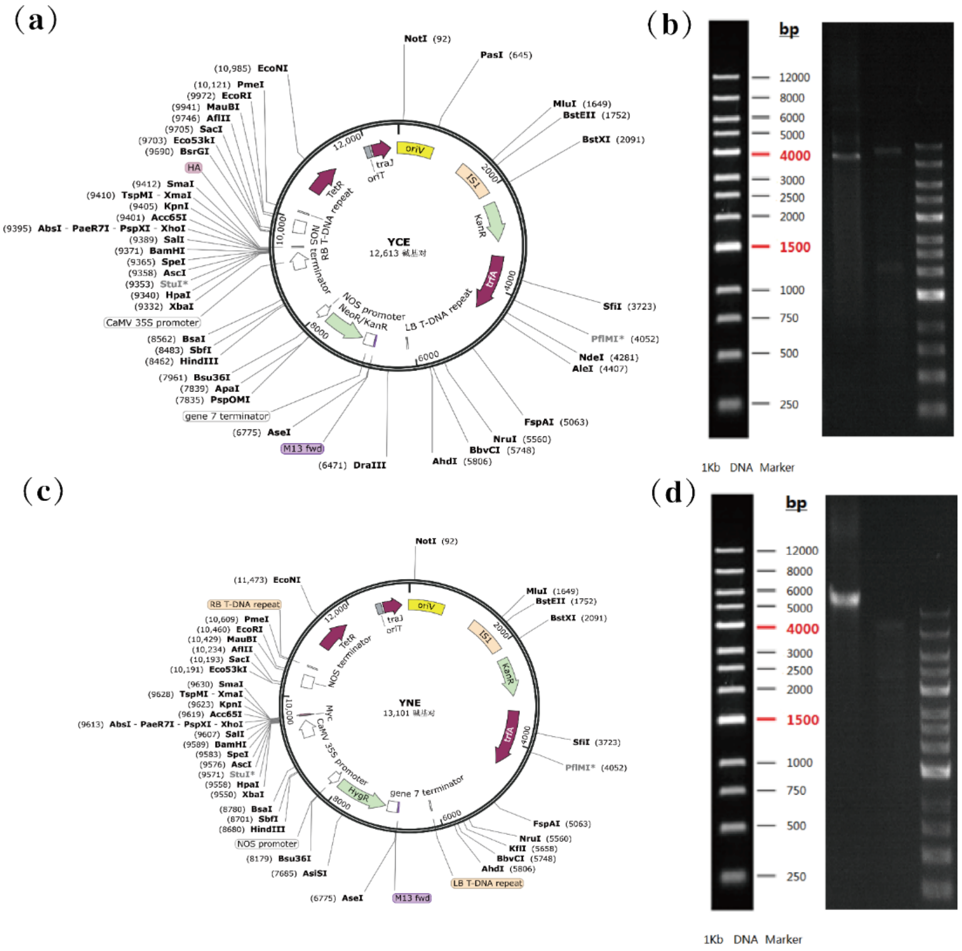

(a) Vector mapping of YCE. (b) Construction of YCE-*PmAGL24* vector. Primers: F-GGAATTCCATGGTGAAAATGATGAGGGAGAAGA; R-CTAGGGAAGCCCCAGTTTGAGAGCAAGCTTG. M is Marker; lane 1 is YCE-*PmAGL24* vector plasmid; lane 2 is enzyme digestion assay (restriction enzyme: *EcoRI-HindIII*). (c) Vector mapping of YNE. (d) Construction of YNE-*PmLHP1* vector. Primers: F-CGGATCCGATGAAAGTGAAGGGAGGAGGAAG; R-TTACAATGTAGAATTGTACCGGTTGCGGCCGCAA. M is Marker; lane 1 is YNE-*PmLHP1* vector plasmid; lane 2 is enzyme digestion assay (restriction enzyme: *BamHI-NotI*).

Supplemental Figure S20. Expression analysis of *AtKNAT2* and *AtkKNAT6* in *lhp1* mutant Arabidopsis.

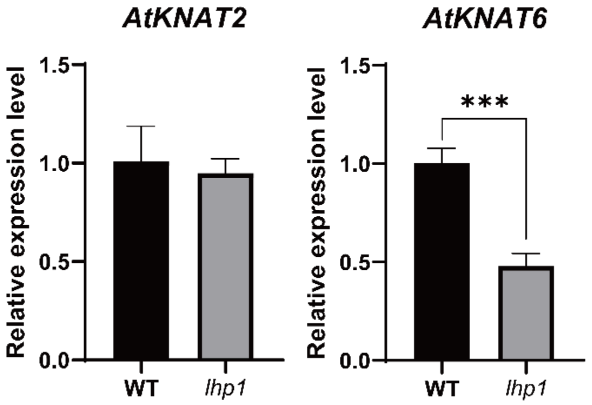

Error bars represent SE based on 3 biological replicates. Asterisks indicate significant differences using Student's *t*-test (***$p < 0.001$; **$p < 0.01$; *$p < 0.05$).

Supplemental Figure S21. Schematic diagram of the ChIP-qPCR primer design region (H3K27me3).

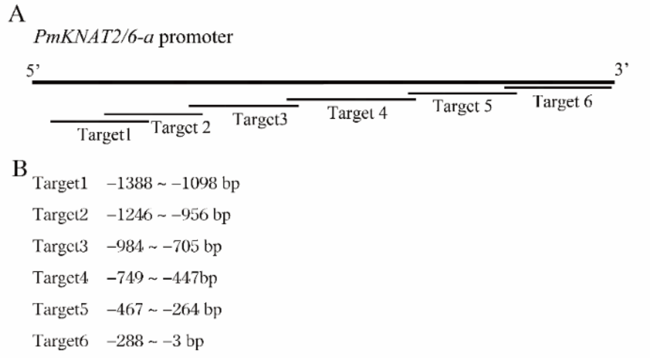

(A) Primer design position diagram. The horizontal line represents the ChIP-qPCR primer design interval. **(B)** Note on the specific location of the promoter of KNAT2/6-a where the primers are located

Supplemental Table S1. Introduction of candidate genes obtained from Y1H screen libraries.

Code	Area of interaction (aa)	Description
NP_001280191.1	6~78	MADS-box protein AGL24-like
XP_008232835.1	3~78	MADS-box protein SOC1
XP_008221062.1	24~230	uncharacterized protein YpgQ-like
XP_008243948.1	193~268	RNA-binding protein CP29B, chloroplastic
XP_008241075.1	119~195	33 kDa ribonucleoprotein, chloroplastic

Supplemental Table S2. Primers design and sequences of Y1H and ChIP-qPCR (PmAGL24).

Genes	Sequences (5′-3′)	
	Forward	Reverse
PmKNAT2/6-a-promoter	GTATGATATGAATGCATAAATGGGCC	CGAGGCCGTACAGTTCCTCCATCAT
PmKNAT2/6-a-pro-F1	GGCCTAAGGTTGTTTTTAATTTGGG	CGAGCCTTGGCTCTGTTCACC
PmKNAT2/6-a-pro-F2	CTCTGGTGAACAGAGCCAAG	GGCCGTACAGTTCCTCCATC
PmKNAT2/6-a-pro-T1	CTCTGGTGAACAGAGCCAAG	GGTTATAAACCCCTTGCTAC
PmKNAT2/6-a-pro-T2	GTAGCAAGGGGTTTATAACC	CATGTATTCCATGCTCGGAG
PmKNAT2/6-a-pro-T3	AGCATTTCTCCGAGCATGGAATAC	GGCCGTACAGTTCCTCCATC

Supplemental Table S3. Predicted functional effects of AGL24 protein point mutations in two Japanese apricot.

Protein name	Site (aa)	Predicted score	Predicted class
AGL24	171	0.000	Neutral
AGL24	184	0.010	Neutral
AGL24	188	0.001	Neutral
AGL24	199	0.013	Neutral
AGL24	200	0.036	Neutral

NOTE: Binary classification of predictions (functional change: score > = 0.5; neutral: score < 0.5)

Supplemental Table S4. Gray values of western blot.

Antibody Name	Band 1	Band 2	Band 3	Marker
H3K27me3	4012.66	5320.78	8955.61	0.00
GAPDH	10796.73	11153.30	10994.08	0.00

Supplemental Table S5. Determination of cytokinin content in flower buds of Japanese apricot at the stage of pistil number differentiation.

Differentiation process	Cultivar	*c*Z	*t*Z	iP
Stage1	DY	0.35 ± 0.03	**3.14 ± 0.07**	0.44 ± 0.04
	LY	0.33 ± 0.01	0.47 ± 0.03	0.33 ± 0.03
Stage2	DY	**0.52 ± 0.05**	**1.68 ± 0.07**	**0.39 ± 0.02**
	LY	0.2 ± 0.02	0.27 ± 0.02	0.18 ± 0.01
Stage3	DY	0.13 ± 0.01	**0.87 ± 0.05**	0.22 ± 0.02
	LY	0.12 ± 0.01	0.36 ± 0.05	0.17 ± 0.02

NOTE: Cytokinins are a class of derivatives of adenine. The synthesis pathway is mainly divided into two types: one is the synthetic pathway, in which the main active substances are *trans*-zeatin (*t*Z) and N6-isopentenyladenine (iP). The other is the catabolic pathway of tRNA, with *cis*-zeatin (*c*Z) as the main active substance. Data with significant differences in ANOVA ($p < 0.05$) in different cultivars are shown in red.

Supplemental Table S6. Primer sequences used for RT-qPCR in Japanese apricot.

**Genes**	**Sequences (5′-3′)**	
	**Forward**	**Reverse**
PmAGL24	TGAATCTGAGGTGGCTGTCG	TGTGGTTTTCATTCTCCAGTTGC
PmLHP1	AGCTCTCGAAGAAGCTGACG	ATACTGGAGCTCACCCTTGC
PmKNAT2/6-a	TGGGAGAAGAGGTTTCGTGC	AAGCATCGAGCTCAGGATCG
PmKNAT2/6-b	ACTTCATCTGCTCACGCCTC	CAAGGTGCCAATGTGACTGC
PmRP2	TGAAGCATACACCTATGATGATGAAG	CTTTGACAGCACCAGTAGATTCC
AtLHP1	TGAAGCCAACAGTGGGAGTG	ACCTTGAGAAGGCCATTGGG
AtAGL24	AGAAGGGCGAGTGTGTGATG	GGGAGTTCCACTGTCGTAGC
AtKNAT2	CAAAACCGATCTTGCGAGGC	TTCAGATCGCGGTCATTGCT
AtKNAT6	TCGATTGCCAAAAGGTCGGA	TTGCCTCGTCAAACGGTCTT
AtActin2	CTGGATTCTGGTGATGGTGTGTCT	GAACCACCGATCCAGACACTGTAC

Supplemental Table S7. Primer sequences used for cloning the CDS regions in Japanese apricot.

Genes	Sequences (5′-3′)	
	Forward	Reverse
PmAGL24	ATGGTGAAAATGATGAGGGAGAAGA	CTAGGGAAGCCCCAGTTTGAGAG
PmLHP1	ATGAAAGTGAAGGGAGGAGGAAG	TTACAATGTAGAATTGTACCGG
PmKNAT2/6-a	ATGATGGAGGAACTGTACGGCC	TCAGTCCTTGGTAAAACAAGGT
PmKNAT2/6-b	ATGGAGGAAATGTACGGATTGC	TCAGTCATCTGTGAAAAATGGA

Supplemental Table S8. Primer sequences used for construction of *atagl24* mutants using the CRISPR/ Cas9 system in Arabidopsis.

Genes	Target sequences (red font)	Primer sequences
Atagl24-F	GAACTCGAAGAGCTTT CCGGTGG	ATATATGGTCTCGATTGAACTCGAAG AGCTTTCCGGGTTTTAGAGCTAGAA
Atagl24-R	ATCCACCTTCTACTCAT CTCCGG	ATTATTGGTCTCGAAACGAGATGAGTA GAAGGTGGACAATCTCTTAGTCGA

Supplemental Table S9. Primer sequences used for ChIP-qPCR in Japanese apricot (H3K27me3).

Targets	Sequences (5′-3′)	
	Forward	Reverse
1	GGCCTAAGGTTGTTTTTAATTTGGG	ATGTGTGTGGGGGCCTCTTA
2	TGTCTATCCCTTTGTCGAGAATAAT	TGCATTGCACCAGTAAGAGC
3	GTGAAAGTGCTCTTACTGGTGC	GAGCCTTGGCTCTGTTCACC
4	CTCTGGTGAACAGAGCCAAG	GGTTATAAACCCCTTGCTAC
5	GTAGCAAGGGGTTTATAACC	CATGTATTCCATGCTCGGAG
6	AGCATTTCTCCGAGCATGGAA	GGCCGTACAGTTCCTCCATC
